# Five-year psychological impact and surveillance compliance in the Australian Pancreatic Cancer Screening Program

**DOI:** 10.1007/s10689-026-00557-0

**Published:** 2026-04-28

**Authors:** Tanya M. Dwarte, David K. E. Chan, Nick Olsen, David B. Williams, Anthony J. Gill, Alina Stoita

**Affiliations:** 1 Australian Pancreatic Cancer Genome Initiative, Garvan Institute of Medical Research, Darlinghurst, NSW Australia; 2 Department of Gastroenterology, St Vincent’s Hospital, 380 Victoria St, Darlinghurst, NSW 2010 Australia; 3 Stats Central, Mark Wainwright Analytical Centre, University of New South Wales, Sydney, NSW Australia; 4 Sydney Medical School, University of Sydney, Sydney, NSW Australia; 5 St Vincent’s Clinical School, University of New South Wales, Sydney, NSW Australia

**Keywords:** Familial pancreatic cancer, Pancreatic cancer surveillance, Psychological impact, Cancer worry, Impact of event scale, Psychological consequences questionnaire

## Abstract

Evidence of benefit for pancreatic ductal adenocarcinoma (PDAC) surveillance is accumulating, including earlier staging, extended survival, and improved psychological function. This study aimed to assess the 5-year psychological impact of high-risk PDAC surveillance, performed at St Vincent’s Hospital, Sydney, Australia. Participants were offered annual endoscopic ultrasound (EUS) or magnetic resonance imaging (MRI), with frequency increasing if clinically indicated. The *impact of event scale* (IES), comprising *intrusion* and *avoidance* subscales, and the *psychological consequences questionnaire* (PCQ), which assesses *emotional*, *physical* and *social* domains, were administered at baseline, 1-month, 1-year and 5-years post-baseline EUS. Generalized linear mixed-effects models were used to assess for differences in reported outcomes. Of the 143 participants under surveillance, 108 underwent annual investigations, and 35 had one or more episode(s) of intensified surveillance. Overall, screening compliance was high, though understandably, increased deferrals occurred during COVID-19 pandemic restrictions. Two participants were diagnosed with pancreatic neoplasms and half had pre-malignant pancreatic lesions that remained stable (n = 45) or showed progression (n = 28). There was a significant reduction in *IES intrusion* and an increase in *positive PCQ emotional, physical* and *total* scores at 5-years. *Negative PCQ* scores remained stable compared to baseline. There was no difference in *IES, negative PCQ* or *positive PCQ* scores based on EUS/MRI findings. Individuals undergoing intensified surveillance reported significantly lower *positive PCQ physical* and *total* scores, but there was no difference in *negative PCQ* or *IES* scores. These data provide reassurance regarding the acceptability and psychological safety of PDAC surveillance, despite frequent abnormal findings and intensified investigation.

**Supplementary Information:**

The online version contains supplementary material available at 10.1007/s10689-026-00557-0.

## Introduction

Early detection of pancreatic ductal adenocarcinoma (PDAC) may improve clinical outcomes. Whilst population screening is not feasible, the Cancer of the Pancreas Screening (CAPS) Consortium recommends surveillance for high-risk individuals [1]. This includes individuals meeting *familial pancreatic cancer* (FPC) criteria, with at least two PDAC-affected first-degree (FDR) and second-degree relatives (SDR) on the same side of the family; those with a clinical or genetic diagnosis of Peutz–Jeghers syndrome (PJS) or hereditary pancreatitis regardless of family history; and carriers of a germline pathogenic variant associated with increased PDAC risk (*BRCA2, ATM, PALB2, CDKN2A, MLH1, PMS2, MSH2* and *MSH6*) who have at least one FDR (or SDR if *BRCA2*) with PDAC.

There is accumulating evidence that PDAC surveillance enables earlier stage detection and improved survival outcomes [2, 3]. Most CAPS consortium sites offer annual EUS and/or magnetic resonance imaging (MRI), with both modalities demonstrating similar detection capabilities and favored as they avoid ionizing radiation exposure [1]. Abnormal pancreatic findings are frequently detected in high-risk cohorts, with cystic lesions most common [4, 5]. In the absence of worrisome or high-risk features, these lesions can typically be safely monitored in accordance with international guidelines [6, 7]. However, individuals with inherited susceptibility demonstrate higher cyst prevalence and increased rates of progression compared with the general population, necessitating ongoing surveillance and, in some cases, intensified follow-up [8]. Multidisciplinary care is required to achieve effective interventions and to reduce patient burdens of intensified surveillance or inappropriate mortality and morbidity [9].

Whilst clinical and survival outcomes remain the primary focus of surveillance programs, their long-term acceptability, psychological safety, and participant adherence are equally critical to program effectiveness [13, 14]. Short-term studies have demonstrated reduction in cancer-related worry and distress following enrolment in high-risk PDAC surveillance programs, including among individuals with abnormal findings or those requiring intensified surveillance [15–17]. Data from the 1-year follow-up from the first 102 participants in our screening program support these findings of positive psychological impacts, irrespective of EUS findings [10]. However, data beyond 1 year is limited despite surveillance being a life-long intervention for most high-risk individuals. Only Konings and colleagues have examined the impact of repeated surveillance at 2–3 years’ follow-up, with a significant reduction in cancer worry and low rates of anxiety and depression at all timepoints [4, 11].

Long-term psychological follow-up is essential to evaluate potential cumulative effects of surveillance, including the burden of repeated investigations and responses to evolving or indeterminate lesions over time. To support transition from research-based surveillance to routine clinical care for high-risk individuals, additional evidence for the long-term sustainability of PDAC surveillance, in terms of compliance, cost-efficiency and psychological safety, is required [12]. Moreover, a deeper understanding of the long-term psychological impacts of high-risk PDAC surveillance may also inform approaches to minimize screening fatigue, which is a common concern in other inherited cancer syndromes [13, 14]. Accordingly, this study extends our previously reported 1-year outcomes to assess 5-year surveillance compliance and long-term psychological impact within the Australian Pancreatic Cancer Screening Program (APCSP), with specific attention to abnormal findings and investigation frequency.

## Methods

The APCSP commenced at St Vincent’s Hospital, Sydney in 2011. Since then, additional sites were established in three other capital cities (Melbourne, Brisbane and Perth). We have previously reported recruitment, genetic counseling, baseline endoscopic ultrasound (EUS) and 1-year psychological outcomes from our screening program [10, 15–17]. Participants enrolled between February 2011 and June 2019 who were eligible for at least 5-years follow-up at the time of analysis (July 2024) were included in this study. Genetic counseling occurred either before or after completion of the baseline questionnaire but before the baseline EUS. Data for each participant were censored at the time of study withdrawal, the 5-year EUS/MRI or 5-year questionnaire completion, whichever occurred last.

### Screening attendance and clinical findings

The standard surveillance protocol was annual EUS (or alternating EUS and MRI) for 5 years. Additional interval investigations (EUS or MRI) were performed at < 1-, 3- or 6-monthly intervals if clinically indicated. Any participant undergoing at least one interval investigation was considered as having ‘intensified’ surveillance, even if they returned to annual surveillance after lesion stability was established. Findings prompting intensified surveillance typically included evolving cystic lesions, interval growth, or features concerning for progression. Due to travel and healthcare restrictions in Australia during the COVID-19 pandemic, only MRI surveillance was offered between March 2020 and March 2022. A participant was considered fully compliant with annual surveillance if they completed an investigation every 12-months (< 4 months allowed for scheduling delays). Non-compliance was recorded if participants deferred a scheduled EUS or did not arrange an MRI using the referral provided.

A “normal” result indicated no observed lesion or irregularity of the pancreatic parenchyma. Results were considered “abnormal” if any of the following features were observed: solid lesion(s); pancreatic cyst(s); intraductal papillary mucinous neoplasm (IPMN) with communication to a side-branch (SB-IPMN) or main pancreatic duct (MD-IPMN); feature(s) indicative of chronic pancreatitis (CP) or duct dilation. Lesion progression was determined by an interval increase in size or number of cystic lesion(s), evolution of communication with a pancreatic duct (e.g. SB-IPMN), worsening CP changes or additional CP features developing. Surveillance findings without high-risk features were explained directly to participants by the study gastroenterologist, and follow-up was conducted in accordance with established guidelines for these lesions. Participants with lesions showing high-risk findings (e.g. MD-IPMN or lesions suspicious for malignancy that underwent biopsy) were reviewed within a multidisciplinary framework. Management strategies for these cases were discussed collaboratively, and results and follow-up plans were subsequently communicated to participants in a structured manner. Additional psychological support was also provided by the study coordinator, who was a certified genetic counselor.

### Psychological assessment

Psychological assessment was a component of the APCSP protocol and was performed throughout the study period via a posted (with a reply-paid envelope enclosed to facilitate return) or emailed questionnaire (Online resource 1–3). At baseline, participants were asked to self-report if they had an existing formal diagnosis of anxiety and/or depression. A 5-point Likert scale was administered at baseline to assess participants’ self-reported worry (1 = not at all worried to 5 = extremely worried) and perceived chance of developing PDAC and other cancer(s) (1 = much below others to 5 = much above others). For FPC participants only, the Bayesian model, PancPRO [18] was used to assess their likelihood of harboring a PDAC predisposition pathogenic variant and estimated lifetime risk for developing PDAC, for comparison with their perceived risk.

Two validated questionnaires, the *impact of event scale (IES)* [19] and *psychological consequences questionnaire (PCQ)* [20] were completed at baseline (pre-intervention), 1-month, 1-year and 5-year post-baseline EUS. In a few cases, the 5-year questionnaire was completed at the time of study withdrawal (3–4 Years follow-up). The IES was used to quantify event-specific distress related to perceived PDAC risk and surveillance participation. For the *IES intrusion* (7 items) and *IES avoidance* (8 items) subscales, responses of “not at all”, “rarely”, “sometimes” and “often” were scored as 0, 1, 3 and 5, respectively, with the *Total IES* score obtained by adding the subscale scores together. Participant raw IES responses were categorized as low, moderate and high, based on subscale ranges of 1–8.5, 8.5–19, and > 19, respectively. The *total IES* scores were categorized as low (0–17), moderate (≥ 17–< 40) and high (≥ 40), with scores greater than 40 indicating a level of distress comparable to thresholds used in post-traumatic stress disorder (PTSD) screening [19]. The PCQ was selected to complement the IES by directly assessing the perceived benefits or harms of cancer surveillance. The PCQ is divided into two components: *negative PCQ* (12 items, measured at all timepoints) and *positive PCQ* (10 items, measured at 1-month; 1-year and 5-years post-baseline EUS), each containing *physical*, *emotional* and *social* domains (subscales), which are added to determine the *total negative PCQ* and *total positive PCQ* scores. Example items on the *negative PCQ* scale included being “unhappy or depressed”, “trouble sleeping”, or “worried about the future”. *Positive PCQ* items included “a sense of reassurance”, “feeling more hopeful”, or “feeling more able to meet home and work responsibilities”.

### Statistical analysis

Mean and standard error (SE) of the reported cancer worry and perceived chance scores as well as raw *IES intrusion*, *IES avoidance* and *total IES* scores, *negative PCQ* and *positive PCQ* subscale and *total* scores were calculated using Microsoft Excel. T-Tests with unequal variances were calculated using Microsoft Excel to assess differences between groups or at each follow-up timepoint compared to baseline, and for those undergoing genetic counseling prior to or after baseline questionnaire completion.

We modelled the subscale and total scores of the *IES, negative PCQ*, and *positive PCQ* over time with independent generalized linear mixed models (GLMMs). A censored normal response distribution was chosen as the observed scores could not be less than 0 or greater than a certain value. The covariates were categorical time, gender, PDAC risk group (FPC/pathogenic variant), EUS/MRI finding (normal/abnormal), and screening frequency (annual/intensified). We controlled for age, personal history (PHx) of anxiety, and PHx of depression. Participant-specific variability was modelled with a participant random intercept nested within a kindred random intercept.

The time-varying covariates (TVCs), EUS/MRI finding and screening frequency, were first time-aligned with the observed scores. We used a between- and within-participant formulation [21] to incorporate them into a GLMM. The within-participant effect measures the change within that individual after an abnormal finding or undergoing intensified surveillance. The between-participant effect is a population-averaged effect, comparing participants based on their average level of the TVC (the percentage of time they had an abnormal EUS finding or underwent intensified surveillance). We fitted the GLMMs using the glmmTMB R package [22] and assessed model assumptions with Dunn-Smyth residual plots [23]. The 95% confidence intervals for the parameter estimates were constructed with 1000 parametric bootstraps. We used estimated marginal means (EMMs) to quantify the effect of time with the emmeans R package [24].

The primary analysis described above was an available case analysis (ACA), where only complete records were analyzed. A secondary analysis that involved multiple imputation (MI) by chained equations, implemented in the mice R package [25], was used to assess the sensitivity of the GLMM parameter estimates under a missing at random assumption because of incomplete records. Thereby, the MI analysis accounts for study withdrawals by estimating missing values, to reduce the bias from study withdrawal. For the ACA, we calculated the subscale score by summing all completed subscale items, and the subscale score was missing if all items within the subscale were incomplete. In the MI analysis, we imputed incomplete item scores then recalculated the subscale scores. In the sensitivity analysis where we followed Cockburn et al.’s [20] imputation, we found consistent inference between the ACA and the sensitivity analysis. In addition to the scores and covariates described above, we included PHx malignancy, the self-reported cancer worry and perceived chance scores, and the participants’ likelihood of having a pathogenic variant in a PDAC susceptibility gene to inform the MI process. We created 15 imputed datasets, based on the average missing data rate [26], to assess the GLMMs described above. Parameter estimates that were significant in both the ACA and MI analyses were deemed robust for inference.

## Results

Data from 143 participants (representing 92 kindreds) were analyzed. Participants’ mean age at baseline was 56 years (range 35–78). The family history of 103 participants met FPC criteria; one had clinically diagnosed PJS but declined genetic testing; and 39 had a germline pathogenic variant with at least one FDR/SDR with PDAC (n = 31 *BRCA2*; n = 7 *PALB2* and n = 1 *MLH1*). Since initial cohort reporting [10], a *PALB2* pathogenic variant was identified in one FPC kindred, with all three participants confirmed to have the familial variant upon predictive testing. Additional participant demographics are summarized in Table 1. Seventy-seven participants attended a genetic counseling appointment prior to completion of the baseline questionnaire. The mean duration of time between genetic counseling and study enrolment/baseline questionnaire completion was 24 months (range 2–169 months). The remaining 66 participants were referred for genetic counseling, with the mean duration of time between baseline questionnaire completion and the counseling appointment 67 days (range 0–357 days).

**Table 1:**
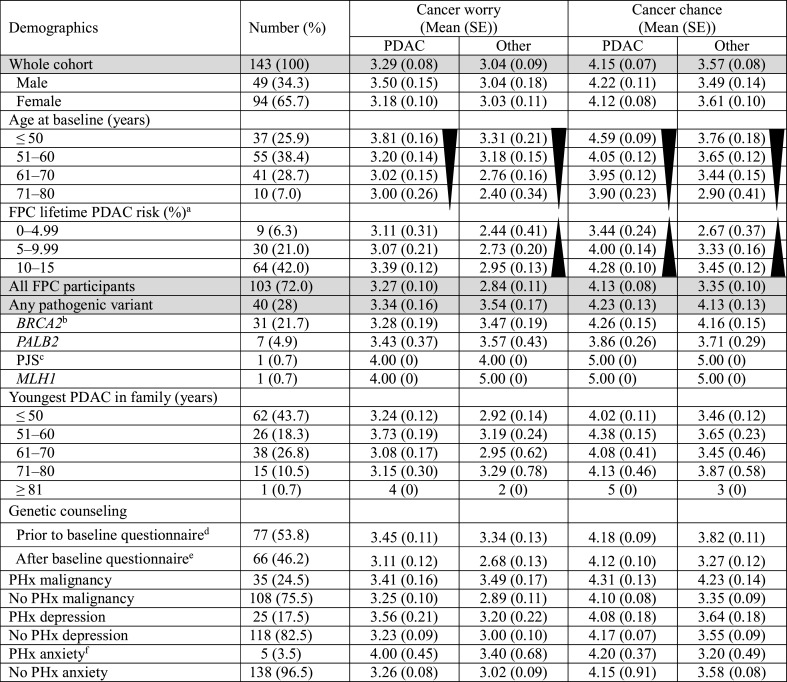
Participant demographics and cancer worry and perceived chance scores at baseline (pre-intervention)

*FPC* familial pancreatic cancer, *PDAC* pancreatic ductal adenocarcinoma, *PHx* personal history, *PJS* Peutz-Jeghers syndrome

^a^Calculated by PancPRO (CancerGene6)

^b^n = 1 *BRCA2* pathogenic variant carrier also had a *BRCA1* pathogenic variant

^c^PJS clinical diagnosis only with no family history of PDAC

^d^All pathogenic variant carriers completed genetic counseling prior to the baseline questionnaire

^e^n = 4 participants completed genetic counseling after the 1-month questionnaire

^f^n = 3 (2.1%) participants reported a personal history of both anxiety and depression

**Fig. 1:**
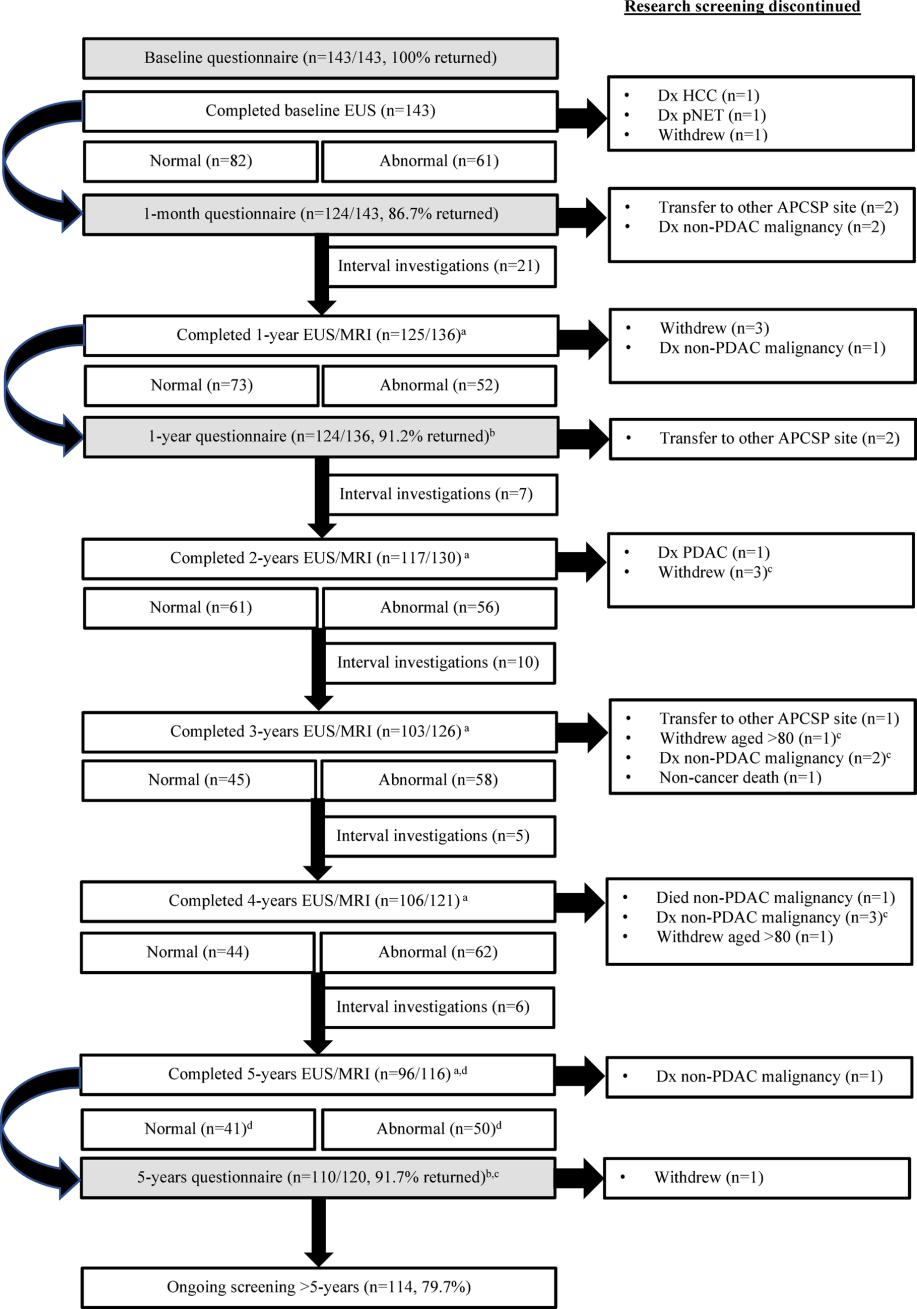
Flowchart of screening attendance, clinical findings, questionnaire completion and study withdrawal. ^a^The denominator for screening investigations performed at each timepoint was adjusted for participant withdrawals and screening deferrals. A higher number of participants with normal clinical findings chose to defer screening during the COVID-19 pandemic. ^b^Some questionnaires were returned during the period of the COVID-19 pandemic (1-year: n = 12 (9.7%); 5-years: n = 24 (21.8%)). ^c^Four participants completed the 5-years questionnaire at the time of study withdrawal. ^d^There were n = 5 screening investigations pending at the time of analysis. *APCSP* Australian Pancreatic Cancer Screening Program, *Dx* diagnosed, *EUS* endoscopic ultrasound, *HCC* hepatocellular carcinoma, *MRI* magnetic resonance imaging, *PDAC* pancreatic ductal adenocarcinoma

Overall screening attendance, clinical findings and questionnaire completion are summarized in Fig. 1. The mean period between baseline questionnaire completion and scheduling of the baseline EUS was 113 days (range 0–351 days) when genetic counseling occurred prior to enrolment, and 142 days (range 2–453 days) for those referred for counseling after enrolment. A total of 734 screening procedures were performed, with a median of six investigations per participant (range 1–11). Sixty-four (44.8%) participants had at least one scheduled screening investigation impacted by COVID-19 restrictions, with n = 12 (9.7%) and n = 24 (21.8%) participants completing the 1-year and 5-year questionnaires, respectively, during the pandemic period.

Seven participants (5.0%) completed a baseline EUS only. Two individuals were diagnosed with a neoplasm on their baseline investigation (n = 1 pancreatic neuroendocrine tumor (pNET); n = 1 hepatocellular carcinoma (HCC)) and two individuals were diagnosed with a non-PDAC malignancy before their 1-year follow-up. Two individuals transferred to another APCSP site, and one participant chose to withdraw from research surveillance.

The remaining 136 participants continued surveillance at a follow-up interval determined by their EUS/MRI findings. Of these, 101 participants (70.6%) continued annual surveillance for the entire study period. More than half were fully compliant with annual surveillance completion for the 5-year period (n = 42) or until study withdrawal (n = 12). Between February 2011 and February 2020, only n = 5 (4.95%) participants assigned to annual surveillance deferred one screening procedure and n = 5 (4.95%) deferred two or more screening procedures. Not surprisingly, during the COVID-19 pandemic, n = 26 (25.7%) and n = 11 (10.9%) participants deferred one or two screening procedures, respectively. Twenty-nine participants assigned to annual surveillance were due for at least one investigation post-COVID-19 (March 2022 to June 2024). Of these, n = 23 (79%) were fully compliant, n = 2 (7%) deferred one screening procedure, and investigations for an additional n = 4 participants were pending at the time of analysis.

Thirty-five participants (24.5%) had at least one interval investigation (intensified surveillance) for a screen-detected abnormality. The nature and progression of these lesions are summarized in Table 2. Only six participants (4.2%) underwent multiple interval investigations. Of those undergoing intensified surveillance, n = 24 (69%) were fully compliant during the 5-year study period. Between 2011 and February 2020, only three participants undergoing intensified surveillance deferred one (n = 2) or two (n = 1) screening procedures. During the COVID-19 pandemic, eight individuals undergoing intensified surveillance deferred one (n = 5) or two (n = 3) screening procedure(s). Only five participants in the intensified surveillance group completed an investigation in the post-COVID-19 period, of which n = 4 were fully compliant, with one investigation pending at the time of analysis. Male participants (34% of the total cohort) showed higher rates of screening deferral(s), accounting for 24% of the fully compliant group, but 47% of both the non-COVID-19 and COVID-19 deferral groups. Pathogenic variant carriers (28% of the total cohort) comprised 27% of the fully compliant group, 13% of non-COVID deferrals and 37% of COVID-deferrals group.

**Table 2:** Endoscopic ultrasound and/or magnetic resonance imaging surveillance findings

Clinical finding	n (%)
Normal (all EUS/MRI)^a^	66 (46.2%)
**Neoplasms detected**^b^	
PDAC	1 (0.7%)
pNET	1 (0.7%)
HCC	1 (0.7%)
**Cyst(s)**^b^	
Stable	14 (9.8%)
Progression	10 (7.0%)
**SB-IPMN(s)**^b^	
Stable	10 (7.0%)
Progression	13 (9.1%)
**Chronic pancreatitis features (e.g. diffuse heterogeneity, strands, foci)**^b^	
Stable	18 (12.5%)
Progression	4 (2.8%)
**Other (e.g. duct dilation, non-specific abnormality)**^b^	
Resolved	1 (0.7%)
Stable	3 (2.1%)
Progression	1 (0.7%)

*EUS* endoscopic ultrasound, *HCC* hepatocellular carcinoma, *MRI* magnetic resonance imaging, *PDAC* pancreatic ductal adenocarcinoma, *pNET* pancreatic neuroendocrine tumor, *SB-IPMN* side-branch intraductal papillary mucinous neoplasm

^a^Up to the time of study withdrawal or at 5-years

^b^If multiple findings were observed in the same participant, they were listed under the more severe feature

Overall, screening acceptability was high with only n = 8 participants (5.6%) electing to discontinue screening during the 5-year study period. Reasons for withdrawal included travel inconvenience (n = 4), participant preference to undergo screening with their local specialist (n = 1) or to defer screening until older (n = 1). Two participants did not indicate a reason for their withdrawal.

One *BRCA2* participant was diagnosed with PDAC on her 2-Year EUS and transferred to clinical management. An additional 19 participants withdrew due to a diagnosis of non-PDAC malignancy (n = 11); non-cancer related death (n = 1), age ≥ 80 years (n = 2) or transfer to an alternate APCSP screening site (n = 5) (Fig. 1).

### Cancer worry and perceived chance scores

At baseline, n = 55 (38.5%) and n = 45 (31.5%) participants reported higher than average or extreme worry about developing PDAC and other cancer(s), respectively. One-hundred and eight (75.5%) and n = 64 (44.8%) participants rated their chance of developing PDAC and other cancer(s), respectively, to be above or much above others. On average, there was reasonable agreement between PancPRO lifetime risk estimates and participants’ perceived PDAC chance scores. FPC participants who were assessed by PancPRO as having moderate (up to 4.99%) lifetime risk reported significantly lower perceived PDAC chance scores, compared to those estimated to have very high risk (10–15%) (3.44 vs. 4.28, *p* = 0.003). This was also observed for the perceived chance of developing other cancer(s) (2.67 vs. 3.45, *p* = 0.027) (Table 1).

### Impact of event scale (IES)

**Fig. 2:**
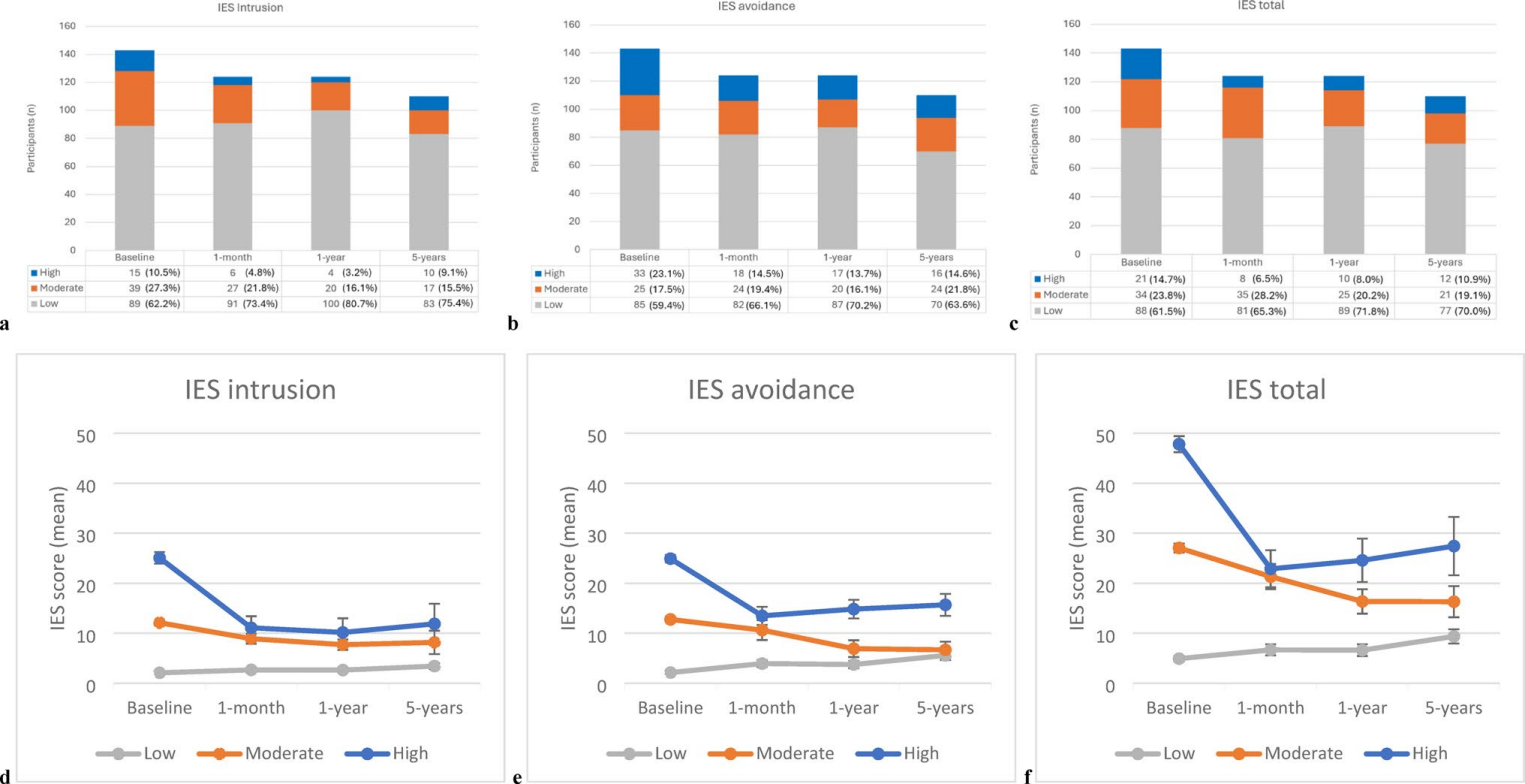
**a**–**c** Number of participants reporting low, moderate and high *Impact of Event (IES) intrusion*, *IES avoidance* and *total IES* scores (raw data). **d**–**f** Mean *IES intrusion*, *IES avoidance* and *total IES* scores over time when categorized as low, moderate or high at baseline. Error bars represent standard error of the mean

At baseline, n = 15 (10.5%) and n = 33 (23%) participants reported high *IES intrusion* and *IES avoidance* scores, respectively, and n = 21 (14.7%) reported a *total IES* score ≥ 40 (Fig. 2a–c). For the *IES intrusion* subscale, the number of participants experiencing moderate and high distress were lower at each follow-up timepoint compared to baseline. For the *IES avoidance* subscale, only the number of participants experiencing high distress showed a reduction over time (Fig. 2b). There were no significant differences in mean baseline *IES intrusion* (*p* = 0.953), *IES avoidance* (*p* = 0.778) or *total IES* (*p* = 0.890) scores between participants completing the baseline questionnaire before or after genetic counseling. There was a significant decrease in the mean *IES intrusion* (*p* < 0.02), *IES avoidance* (*p* < 0.01) and *total IES* scores (*p* < 0.01) at all follow-up timepoints for participants reporting high distress at baseline (Fig. 2d–f). In the moderate distress group, there was a significant decrease in the mean *IES intrusion* (*p* < 0.02), and *total IES* scores (*p* < 0.04), at all follow-up time points, and a significant reduction at 1-year (*p* = 0.003) and 5-year (*p* < 0.001) *IES avoidance* scores compared to baseline. There was a significant increase in *IES avoidance* scores (*p* < 0.05) in the low distress group at all timepoints and an increase in *IES intrusion* (*p* = 0.035) and *IES total* scores at 5-years (*p* = 0.004), though on average these remained in the low distress range (< 8.5 for subscale and < 17 for total score).

The EMMs (represented as $\widehat{\mu }$) and the GLMM parameter estimates for the ACA and MI analyses of the *IES intrusion*, *IES avoidance*, and t*otal IES* scores are displayed in Tables 3 and 4, respectively. The interpretation of the parameter estimates is in terms of adjusted effects. For completeness, the EMMs on the uncensored scale can be found in Online resource 4.

**Table 3:** Estimated marginal means for *impact of event scale* scores using generalized linear mixed model analyses

Impact of event scale	Estimated marginal mean $\widehat{\mu }$ (95% HPD CI)	
	Available case analysis	Multiple imputation
**Intrusion**		
Baseline	8.19 (4.59–11.83)	7.69 (4.20–11.17)
1-month	5.02 (0.61–8.25)	4.07 (0.53–7.62)
1-year	5.46 (1.29–8.80)	3.86 (0.32–7.39)
5-years	6.32 (2.13–9.87)	4.23 (0.67–7.80)
**Avoidance**		
Baseline	9.22 (3.68–14.11)	8.75 (3.87–13.63)
1-month	6.19 (0.73–11.20)	5.29 (0.37–10.21)
1-year	6.58 (1.12–11.93)	4.65 (0.00–9.56)^a^
5-years	8.58 (2.95–13.70)	5.59 (0.62–10.56)
**Total**		
Baseline	18.65 (9.68–25.60)	17.72 (10.48–24.96)
1-month	12.56 (4.32–20.16)	10.96 (3.66–18.27)
1-year	13.58 (5.29–20.90)	10.35 (3.07–17.63)
5-years	16.17 (7.73–23.64)	11.57 (4.22–18.92)

*CI* confidence interval, *HPD* highest posterior density

^a^95% HDP CI exceeded the lower limit

**Table 4:** Summary of *impact of event scale* scores using generalized linear mixed model analyses

Impact of event scale	GLMM parameter estimates			
	Available case analysis		Multiple imputation	
	$\widehat{\beta }$ (95% CI)	*p* value	$\widehat{\beta }$ (95% CI)	*p* value
**Intrusion**				
1-month	− 3.17 (− 4.66 to − 1.57)	**< 0.001**	− 3.61 (− 5.19 to − 2.04)	**< 0.001**
1-year	− 2.73 (− 4.37 to − 1.21)	**< 0.001**	− 3.83 (− 5.39 to − 2.27)	**< 0.001**
5-years	− 1.87 (− 3.70 to − 0.08)	**0.034**	− 3.45 (− 5.19 to − 1.71)	**< 0.001**
**Avoidance**				
1-month	− 3.03 (− 5.36 to − 0.94)	**0.004**	− 3.46 (− 5.51 to − 1.41)	**< 0.001**
1-year	− 2.65 (− 4.99 to − 0.45)	**0.030**	− 4.10 (− 6.22 to − 1.98)	**< 0.001**
5-years	− 0.65 (− 3.07 to 1.87)	0.630	− 3.17 (− 5.48 to − 0.85)	**0.008**
**Total**				
1-month	− 6.09 (− 9.47 to − 2.91)	**< 0.001**	− 6.76 (− 9.85 to − 3.66)	**< 0.001**
1-year	− 5.07 (− 8.52 to − 2.08)	**0.002**	− 7.37 (− 10.50 to − 4.23)	**< 0.001**
5-years	− 2.48 (− 6.30 to 1.26)	0.210	− 6.15 (− 9.59 to − 2.70)	**< 0.001**
**Age (per year of age)**				
Intrusion	− 0.18 (− 0.31 to − 0.03)	**0.010**	− 0.15 (− 0.27 to − 0.02)	**0.028**
Avoidance	− 0.33 (− 0.53 to − 0.14)	**0.002**	− 0.26 (− 0.44 to − 0.08)	**0.004**
Total	− 0.46 (− 0.78 to − 0.15)	**0.004**	− 0.36 (− 0.63 to − 0.10)	**0.008**
**Female**				
Intrusion	− 1.02 (− 4.08 to 1.60)	0.512	− 0.55 (− 3.14 to 2.03)	0.675
Avoidance	− 1.17 (− 4.82 to 2.42)	0.534	− 0.31 (− 3.87 to 3.25)	0.864
Total	− 2.55 (− 8.31 to 3.84)	0.426	− 1.29 (− 6.61 to 4.03)	0.633
**FPC**				
Intrusion	1.43 (− 1.42 to 4.25)	0.374	1.27 (− 1.43 to 3.97)	0.356
Avoidance	− 0.14 (− 4.19 to 4.04)	0.952	− 0.45 (− 4.20 to 3.31)	0.815
Total	1.24 (− 5.05 to 7.53)	0.702	0.83 (− 4.76 to 6.41)	0.772
**Abnormal EUS/MRI (between-participant)**				
Intrusion	1.42 (− 1.62 to 4.50)	0.398	0.73 (− 2.11 to 3.58)	0.613
Avoidance	2.11 (− 2.29 to 6.38)	0.386	0.90 (− 3.07 to 4.86)	0.657
Total	3.50 (− 2.73 to 9.69)	0.270	1.64 (− 4.25 to 7.53)	0.585
**Abnormal EUS/MRI (within-participant)**				
Intrusion	1.61 (− 2.11 to 4.97)	0.388	2.02 (− 1.68 to 5.73)	0.282
Avoidance	0.61 (− 4.05 to 5.23)	0.836	2.12 (− 2.44 to 6.68)	0.361
Total	2.51 (− 5.23 to 10.20)	0.516	3.93 (− 3.16 to 11.02)	0.275
**Intensified (between-participant)**				
Intrusion	1.37 (− 8.48 to 10.61)	0.754	3.78 (− 3.96 to 11.51)	0.338
Avoidance	1.64 (− 11.07 to 14.59)	0.760	5.10 (− 5.62 to 15.82)	0.350
Total	2.61 (− 15.85 to 22.03)	0.746	8.01 (− 8.05 to 24.06)	0.328
**Intensified (within-participant)**				
Intrusion	2.29 (− 0.51 to 5.06)	0.140	0.87 (− 2.07 to 3.81)	0.561
Avoidance	2.32 (− 1.30 to 6.08)	0.284	− 0.09 (− 3.95 to 3.77)	0.964
Total	4.29 (− 1.44 to 9.95)	0.124	0.81 (− 4.92 to 6.55)	0.780
**PHx depression**				
Intrusion	5.41 (1.87 to 8.74)	**0.010**	3.91 (0.70 to 7.12)	**0.017**
Avoidance	6.67 (1.77 to 11.19)	**0.006**	4.81 (0.39 to 9.24)	**0.033**
Total	11.21 (3.90 to 18.65)	**0.006**	7.87 (1.23 to 14.51)	**0.020**
**PHx anxiety**				
Intrusion	1.40 (− 5.41 to 8.37)	0.684	1.16 (− 5.40 to 7.73)	0.728
Avoidance	0.10 (− 10.52 to 9.87)	0.992	− 0.18 (− 9.43 to 9.06)	0.969
Total	1.64 (− 14.10 to 16.57)	0.876	1.30 (− 12.38 to 14.99)	0.852

Significant differences (*p* values < 0.05) are in bold

*CI* confidence interval, *EUS* endoscopic ultrasound, *FPC* familial pancreatic cancer, *HPD* highest posterior density, *MRI* magnetic resonance imaging, *PDAC* pancreatic ductal adenocarcinoma, *PHx* personal history

In the ACA, the average *IES intrusion* score over time was lower compared to baseline [baseline $\widehat{\mu }$ = 8.19; 1-month, $\widehat{\mu }$ = 5.02, $\widehat{\beta }$ = − 3.17, *p* < 0.001; 1-year, $\widehat{\mu }$ = 5.46, $\widehat{\beta }$ = − 2.73, *p* < 0.001; 5-years, $\widehat{\mu }$ = 6.32, $\widehat{\beta }$ = − 1.87, *p* = 0.034 (Tables 3 and 4)]. Furthermore, the average *IES avoidance* scores at 1-month [$\widehat{\mu }$ = 6.19, $\widehat{\beta }$ = − 3.03, *p* = 0.004] and 1-year [$\widehat{\mu }$ = 6.58, $\widehat{\beta }$ = − 2.65, *p* = 0.030] were lower compared to baseline [$\widehat{\mu }$ = 9.22], and likewise for the average *Total IES* score [baseline $\widehat{\mu }$ = 18.65; 1-month, $\widehat{\mu }$ = 12.56, $\widehat{\beta }$ = − 6.09, *p* < 0.001; 1-year, $\widehat{\mu }$ = 13.58, $\widehat{\beta }$ = − 5.07, *p* = 0.002]. The preceding results were consistent with our MI analysis. There was no difference in the average *IES avoidance* and *total IES* scores at 5-years compared to baseline in the ACA. Yet, in the MI analysis, the average *IES avoidance* [${\widehat{\mu }}_{MI}$ = 5.59, ${\widehat{\beta }}_{MI}$ = − 3.17, *p* = 0.008] and *total IES* [${\widehat{\mu }}_{MI}$ = 11.57, ${\widehat{\beta }}_{MI}$ = − 6.15, *p* < 0.001] scores were estimated to be lower at 5-years compared to baseline (Tables 3 and 4).

In the ACA, the average *IES intrusion* [$\widehat{\beta }$ = − 0.18, *p* = 0.010], *IES avoidance* [$\widehat{\beta }$ = − 0.33, *p* = 0.002], and *total IES* [$\widehat{\beta }$ = − 0.46, *p* = 0.004] scores decreased as age at baseline increases (Table 4). Participants with PHx depression had an elevated average *IES intrusion* [$\widehat{\beta }$ = 5.41, *p* = 0.010], *IES avoidance* [$\widehat{\beta }$ = 6.67, *p* = 0.006], and *total IES* score [$\widehat{\beta }$ = 11.21, *p* = 0.006] compared to those without (Table 4). There were no other covariates that were significantly associated with the *IES* scores, including EUS/MRI findings and surveillance frequency. These results were consistent with our MI analysis.

### Psychological consequences questionnaire (PCQ)

There were no significant differences in mean baseline *negative PCQ* subscale and *total* scores (*emotional*: *p* = 0.566; *physical:* *p* = 0.957; *social:* *p* = 0.490; and *total:* *p* = 0.646), between participants completing the baseline questionnaire before or after genetic counseling. *Positive PCQ* scores were first administered at the 1-month timepoint, at which time all participants had completed their baseline EUS and a genetic counseling appointment. Whilst the mean duration of time since genetic counseling differed (24 months vs. 67 days, *p* < 0.0001), there were no significant differences in the mean 1-month *positive PCQ* subscale and *total* scores (*emotional*: *p* = 0.514; *physical:* *p* = 0.440; *social:* *p* = 0.493; and *total:* *p* = 0.952), between participants who completed genetic counseling before or after study enrolment. The EMMs and the GLMM parameter estimates for the ACA and MI analyses of the *negative PCQ* and *positive PCQ* subscales and *total* scores are displayed in Tables 5 and 6, respectively. Likewise, the interpretation of the parameter estimates is in terms of adjusted effects. For completeness, the EMMs on the uncensored scale can be found in Online resource 4.

**Table 5:** Estimated marginal means for negative and positive *psychological consequences questionnaire* scores using generalized linear mixed model analyses

Psychological consequences questionnaire	Estimated marginal mean $\widehat{\mu }$ (95% HPD CI)			
	Negative PCQ		Positive PCQ	
	Available case analysis	Multiple imputation	Available case analysis	Multiple imputation
**Emotional**				
Baseline	1.36 (0.00–3.61)^a^	1.48 (0.00–3.47)^a^	–	–
1-month	0.60 (0.00–2.41)^a^	0.40 (0.00–2.43)^a^	10.97 (8.28–13.99)	10.82 (8.67–12.97)
1-year	0.88 (0.00–3.01)^a^	0.26 (0.00–2.29)^a^	12.11 (9.40–15.00)^c^	12.08 (9.93–14.23)
5-years	1.25 (0.00–3.40)^a^	0.07 (0.00–2.11)^a^	13.84 (11.06–15.00)^c^	13.35 (11.12–15.00)^c^
**Physical**				
Baseline	0.00 (0.00–1.09)^b^	0.00 (0.00–0.63)^b^	–	–
1-month	0.00 (0.00–0.48)^b^	0.00 (0.00–0.00)^b^	3.38 (0.00–7.14)^a^	3.23 (0.32–6.15)
1-year	0.00 (0.00–0.53)^b^	0.00 (0.00–0.00)^b^	3.33 (0.00–7.18)^a^	3.37 (0.46–6.28)
5-years	0.00 (0.00–1.25)^b^	0.00 (0.00–0.00)^b^	5.81 (1.89–9.00)^c^	5.89 (2.92–8.87)
**Social**				
Baseline	0.00 (0.00–0.24)^b^	0.00 (0.00–0.16)^b^	–	–
1-month	0.00 (0.00–0.13)^b^	0.00 (0.00–0.00)^b^	1.69 (0.00–4.10)^a^	1.72 (0.00–3.77)^a^
1-year	0.00 (0.00–0.59)^b^	0.00 (0.00–0.00)^b^	1.72 (0.00–4.35)^a^	1.95 (0.00–4.01)^a^
5-years	0.00 (0.00–0.98)^b^	0.00 (0.00–0.00)^b^	3.35 (1.03–5.98)	3.60 (1.54–5.66)
**Total**				
Baseline	2.85 (0.00–7.00)^a^	3.06 (0.00–6.81)^a^	–	–
1-month	1.44 (0.00–5.62)^a^	1.04 (0.00–4.81)^a^	18.28 (14.03–23.14)	17.57 (13.73–21.41)
1-year	2.04 (0.00–7.08)^a^	1.06 (0.00–4.85)^a^	19.27 (14.89–23.78)	18.82 (14.95–22.68)
5-years	3.08 (0.00–6.94)^a^	0.52 (0.00–4.38)^a^	23.27 (18.62–28.00)	22.27 (18.35–26.18)

^a^95% HDP CI exceeded the lower limit

^b^Estimated marginal mean and 95% HPD CI exceeded the lower limit

^c^95% HPD CI exceeded the upper limit

*CI* confidence interval, *EUS* endoscopic ultrasound, *FPC* familial pancreatic cancer, *HPD* highest posterior density, *MRI* magnetic resonance imaging, *PDAC* pancreatic ductal adenocarcinoma, *PHx* personal history

**Table 6:** Summary of negative and positive *psychological consequences questionnaire* scores using generalized linear mixed model analyses

Psychological consequences questionnaire	GLMM parameter estimates							
	Negative PCQ				Positive PCQ			
	Available case analysis		Multiple imputation		Available case analysis		Multiple imputation	
	$\widehat{\beta }$ (95% CI)	*p* value	$\widehat{\beta }$ (95% CI)	*p* value	$\widehat{\beta }$ (95% CI)	*p* value	$\widehat{\beta }$ (95% CI)	*p* value
**Emotional**								
1-month	− 0.76 (− 1.74 to 0.19)	0.090	− 1.07 (− 2.02 to − 0.13)	**0.027**		–	–	–
1-year	− 0.49 (− 1.47 to 0.47)	0.308	− 1.21 (− 2.19 to − 0.24)	**0.015**	1.14 (0.08 to 2.24)	**0.034**	1.26 (0.29 to 2.23)	**0.011**
5-years	− 0.11 (− 1.17 to 1.00)	0.866	− 1.41 (− 2.47 to − 0.34)	**0.010**	2.86 (1.62 to 4.23)	**< 0.001**	2.53 (1.39 to 3.68)	**< 0.001**
**Physical**								
1-month	− 0.64 (− 1.64 to 0.28)	0.162	− 1.06 (− 2.08 to − 0.05)	**0.040**		–	–	–
1-year	− 0.40 (− 1.44 to 0.60)	0.482	− 1.01 (− 2.04 to 0.03)	0.056	− 0.05 (− 1.62 to 1.54)	0.910	0.13 (− 1.24 to 1.51)	0.847
5-years	0.23 (− 1.00 to 1.33)	0.722	− 1.23 (− 2.38 to − 0.09)	**0.035**	2.43 (0.71 to 4.20)	**0.004**	2.66 (1.09 to 4.23)	**0.001**
**Social**								
1-month	− 0.32 (− 1.27 to 0.68)	0.568	− 0.63 (− 1.63 to 0.36)	0.212		–	–	–
1-year	0.13 (− 0.77 to 1.17)	0.764	− 0.34 (− 1.35 to 0.66)	0.501	0.02 (− 0.96 to 1.05)	0.926	0.23 (− 0.78 to 1.24)	0.651
5-years	0.57 (− 0.52 to 1.65)	0.308	− 0.91 (− 2.06 to 0.24)	0.119	1.66 (0.47 to 2.85)	**0.008**	1.88 (0.75 to 3.02)	**0.001**
**Total**								
1-month	− 1.41 (− 3.05 to 0.31)	0.106	− 2.02 (− 3.72 to − 0.32)	**0.020**		–	–	–
1-year	− 0.82 (− 2.47 to 0.95)	0.358	− 2.00 (− 3.74 to − 0.25)	**0.025**	0.99 (− 0.83 to 2.79)	0.322	1.24 (− 0.33 to 2.81)	0.120
5-years	0.22 (− 1.82 to 2.23)	0.826	− 2.54 (− 4.47 to − 0.61)	**0.010**	4.99 (2.82 to 7.17)	**< 0.001**	4.69 (2.93 to 6.46)	**< 0.001**
**Age (per year of age)**								
Emotional	− 0.10 (− 0.17 to − 0.02)	**0.010**	− 0.09 (− 0.17 to − 0.02)	**0.013**	− 0.08 (− 0.16 to 0.02)	0.122	− 0.09 (− 0.17 to − 0.01)	**0.028**
Physical	− 0.07 (− 0.15 to 0.00)	0.054	− 0.05 (− 0.12 to 0.02)	0.170	− 0.12 (− 0.24 to − 0.01)	**0.044**	− 0.10 (− 0.21 to 0.01)	0.063
Social	− 0.11 (− 0.18 to − 0.04)	**0.002**	− 0.11 (− 0.18 to − 0.03)	**0.004**	− 0.07 (− 0.15 to 0.01)	0.072	− 0.06 (− 0.14 to 0.01)	0.106
Total	− 0.20 (− 0.37 to − 0.05)	**< 0.001**	− 0.18 (− 0.32 to − 0.04)	**0.014**	− 0.15 (− 0.31 to 0.00)	0.068	− 0.15 (− 0.29 to − 0.01)	**0.033**
**Female**								
Emotional	− 0.75 (− 2.34 to 0.73)	0.304	− 0.40 (− 1.80 to 1.00)	0.571	1.03 (− 0.82 to 2.79)	0.258	0.82 (− 0.70 to 2.35)	0.289
Physical	− 1.34 (− 2.77 to 0.06)	0.064	− 0.85 (− 2.24 to 0.54)	0.231	0.26 (− 2.06 to 2.59)	0.796	0.59 (− 1.56 to 2.74)	0.590
Social	− 1.37 (− 2.69 to − 0.06)	**0.032**	− 1.01 (− 2.30 to 0.29)	0.127	0.25 (− 1.43 to 1.84)	0.802	0.42 (− 1.11 to 1.95)	0.587
Total	− 2.16 (− 5.12 to 0.77)	0.166	− 1.27 (− 3.90 to 1.37)	0.346	1.22 (− 1.90 to 4.06)	0.456	1.31 (− 1.46 to 4.08)	0.353
**FPC**								
Emotional	− 0.08 (− 1.62 to 1.54)	0.934	− 0.19 (− 1.67 to 1.30)	0.805	1.28 (− 0.73 to 3.36)	0.198	0.90 (− 0.76 to 2.55)	0.289
Physical	0.62 (− 0.94 to 2.10)	0.390	0.43 (− 1.06 to 1.93)	0.569	2.66 (0.29 to 5.35)	**0.028**	1.20 (− 1.06 to 3.46)	0.296
Social	0.49 (− 0.92 to 2.11)	0.512	0.49 (− 0.98 to 1.95)	0.515	1.84 (0.10 to 3.73)	**0.034**	1.00 (− 0.60 to 2.60)	0.221
Total	0.16 (− 2.81 to 3.54)	0.904	− 0.03 (− 2.89 to 2.82)	0.981	2.70 (− 0.31 to 5.98)	0.090	1.71 (− 1.17 to 4.59)	0.243
**Abnormal EUS/MRI (between-participant)**								
Emotional	0.52 (− 1.19 to 2.19)	0.586	− 0.08 (− 1.64 to 1.48)	0.918	− 1.99 (− 3.99 to 0.05)	0.058	− 1.09 (− 2.81 to 0.63)	0.215
Physical	0.47 (− 1.20 to 2.18)	0.586	0.01 (− 1.58 to 1.59)	0.995	− 1.19 (− 3.89 to 1.56)	0.356	− 0.29 (− 2.72 to 2.15)	0.816
Social	− 0.10 (− 1.63 to 1.45)	0.860	− 0.48 (− 1.98 to 1.02)	0.526	− 1.15 (− 2.93 to 0.54)	0.186	− 0.42 (− 2.13 to 1.29)	0.630
Total	1.10 (− 2.26 to 4.25)	0.518	− 0.09 (− 3.08 to 2.90)	0.954	− 2.25 (− 5.72 to 0.95)	0.158	− 1.12 (− 4.18 to 1.93)	0.470
**Abnormal EUS/MRI (within-participant)**								
Emotional	0.26 (− 2.07 to 2.43)	0.782	1.07 (− 0.92 to 3.06)	0.290	− 2.36 (− 4.83 to 0.28)	0.088	− 1.45 (− 3.86 to 0.95)	0.232
Physical	0.79 (− 1.24 to 2.96)	0.484	1.62 (− 0.49 to 3.72)	0.132	1.63 (− 2.14 to 5.10)	0.382	1.00 (− 2.19 to 4.18)	0.536
Social	− 0.13 (− 2.29 to 2.04)	0.902	0.64 (− 1.33 to 2.61)	0.525	1.40 (− 0.86 to 4.03)	0.244	0.92 (− 1.38 to 3.21)	0.429
Total	0.80 (− 3.32 to 4.95)	0.670	2.48 (− 1.20 to 6.15)	0.186	0.01 (− 4.27 to 4.74)	0.978	0.37 (− 3.39 to 4.14)	0.845
**Intensified (between-participant)**								
Emotional	0.57 (− 4.00 to 5.15)	0.816	2.25 (− 1.99 to 6.48)	0.298	2.39 (− 3.08 to 8.28)	0.416	1.00 (− 3.61 to 5.61)	0.670
Physical	− 0.53 (− 5.49 to 4.01)	0.824	0.59 (− 3.70 to 4.87)	0.788	3.17 (− 3.54 to 10.10)	0.396	1.76 (− 4.70 to 8.23)	0.592
Social	0.31 (− 4.61 to 4.41)	0.980	1.76 (− 2.13 to 5.64)	0.375	2.52 (− 2.50 to 7.53)	0.310	1.64 (− 2.93 to 6.22)	0.481
Total	0.28 (− 8.98 to 9.84)	0.964	3.33 (− 4.67 to 11.33)	0.413	3.60 (− 5.81 to 14.17)	0.446	2.08 (− 6.30 to 10.45)	0.626
**Intensified (within-participant)**								
Emotional	1.35 (− 0.23 to 2.96)	0.098	0.44 (− 1.31 to 2.18)	0.622	− 2.06 (− 4.22 to 0.26)	0.090	− 1.88 (− 3.99 to 0.23)	0.081
Physical	0.43 (− 1.22 to 2.26)	0.628	− 0.46 (− 2.40 to 1.47)	0.637	− 4.31 (− 7.66 to − 1.43)	**0.002**	− 3.37 (− 6.49 to − 0.26)	**0.034**
Social	1.59 (− 0.15 to 3.35)	0.062	0.58 (− 1.25 to 2.41)	0.533	− 1.89 (− 4.08 to 0.05)	0.058	− 1.20 (− 3.48 to 1.07)	0.297
Total	2.32 (− 0.94 to 5.28)	0.138	0.14 (− 3.01 to 3.30)	0.928	− 4.00 (− 7.69 to − 0.62)	**0.024**	− 3.51 (− 6.87 to − 0.15)	**0.041**
**PHx depression**								
Emotional	3.41 (1.54 to 5.41)	**0.002**	2.45 (0.67 to 4.24)	**0.007**	− 0.30 (− 2.56 to 2.24)	0.816	0.28 (− 1.69 to 2.25)	0.781
Physical	3.09 (1.28 to 5.04)	**< 0.001**	2.31 (0.53 to 4.08)	**0.011**	2.44 (− 0.44 to 5.42)	0.100	2.44 (− 0.22 to 5.09)	0.072
Social	2.14 (0.47 to 3.78)	**0.016**	1.63 (0.00 to 3.26)	0.050	1.31 (− 0.68 to 3.07)	0.196	1.53 (− 0.33 to 3.39)	0.107
Total	6.14 (2.44 to 10.33)	**< 0.001**	4.12 (0.70 to 7.53)	**0.018**	2.24 (− 1.32 to 6.31)	0.252	2.59 (− 0.91 to 6.08)	0.147
**PHx anxiety**								
Emotional	− 2.58 (− 7.21 to 1.21)	0.176	− 1.93 (− 5.69 to 1.83)	0.314	2.68 (− 1.81 to 8.84)	0.274	2.13 (− 2.03 to 6.30)	0.315
Physical	− 2.51 (− 44.56 to 1.29)	0.194	− 2.02 (− 6.14 to 2.10)	0.336	3.30 (− 2.78 to 10.49)	0.274	3.68 (− 1.85 to 9.21)	0.192
Social	− 2.58 (− 20.10 to 0.62)	0.112	− 2.09 (− 5.90 to 1.71)	0.280	2.08 (− 1.95 to 6.90)	0.308	2.72 (− 1.19 to 6.62)	0.173
Total	− 4.14 (− 13.90 to 3.92)	0.316	− 2.97 (− 9.99 to 4.05)	0.407	7.11 (− 1.42 to 16.36)	0.098	6.04 (− 1.41 to 13.49)	0.112

Significant differences (*p* values < 0.05) are in bold

*CI* confidence interval, *EUS* endoscopic ultrasound, *FPC* familial pancreatic cancer, *HPD* highest posterior density, *MRI* magnetic resonance imaging, *PDAC* pancreatic ductal adenocarcinoma, *PHx* personal history

### Negative PCQ

In the ACA, there was no difference in the average *negative PCQ emotional*, *physical*, *social*, and *total* scores over time when compared to baseline (Table 5). However, in the MI analysis, the average *negative PCQ emotional* and *total* scores at 1-month [*emotional*: ${\widehat{\mu }}_{MI}$ = 0.40, ${\widehat{\beta }}_{MI}$ = − 1.07, *p* = 0.027; *total*: ${\widehat{\mu }}_{MI}$ = 1.04, ${\widehat{\beta }}_{MI}$ = − 2.02, *p* = 0.020], 1-year [*emotional*: ${\widehat{\mu }}_{MI}$ = 0.26, ${\widehat{\beta }}_{MI}$ = − 1.21, *p* = 0.015; *total*: ${\widehat{\mu }}_{MI}$ = 1.06, ${\widehat{\beta }}_{MI}$ = − 2.00, *p* = 0.025], and 5-years [*emotional*: ${\widehat{\mu }}_{MI}$ = 0.07, ${\widehat{\beta }}_{MI}$ = − 1.41, *p* = 0.010; *total*: ${\widehat{\mu }}_{MI}$ = 0.52, ${\widehat{\beta }}_{MI}$ = − 2.54, *p* = 0.010] were lower compared to baseline [*emotional*: ${\widehat{\mu }}_{MI}$ = 1.48; *total*: ${\widehat{\mu }}_{MI}$ = 3.06] (Tables 5 and 6). Furthermore, in our MI analysis, the average *negative PCQ physical* scores at 1-month [${\widehat{\mu }}_{MI}$ = 0.00, ${\widehat{\beta }}_{MI}$ = − 1.06, *p* = 0.040] and 5-years [${\widehat{\mu }}_{MI}$ = 0.00, ${\widehat{\beta }}_{MI}$ = − 1.23, *p* = 0.035] were lower compared to baseline [${\widehat{\mu }}_{MI}$ = 0.00]. These differences ($\widehat{\beta }$) over time were estimated on the uncensored scale of the *negative PCQ* scores, due to using a censored normal response distribution.

In the ACA, the average *negative PCQ emotional* [$\widehat{\beta }$ = − 0.10, *p* = 0.010], *social* [$\widehat{\beta }$ = − 0.11, *p* = 0.002], and *total* [$\widehat{\beta }$ = − 0.20, *p* < 0.001] scores decreased as age at baseline increases (Table 6). This was consistent with the MI analysis. Participants with PHx depression had an elevated average *negative PCQ emotional* [$\widehat{\beta }$ = 3.41, *p* = 0.002], *physical* [$\widehat{\beta }$ = 3.09, *p* < 0.001], *social* [$\widehat{\beta }$ = 2.14, *p* = 0.016], and *total* [$\widehat{\beta }$ = 6.14, *p* < 0.001] score compared to those who did not (Table 6). However, this effect for the *negative PCQ social* score was not replicated in our MI analysis. Similarly, the ACA showed that female participants had a lower *negative PCQ social* score [$\widehat{\beta }$ = − 1.37, *p* = 0.032], but this effect was not replicated in the MI analysis (Table 6). There were no other covariates that were significantly associated with the *negative PCQ* scores, including EUS/MRI findings and surveillance frequency.

### Positive PCQ

In the ACA, the average *positive PCQ emotional* score at 1-year [$\widehat{\mu }$ = 12.11, $\widehat{\beta }$ = 1.14, *p* = 0.034] and 5-years [$\widehat{\mu }$ = 13.84, $\widehat{\beta }$ = 2.86, *p* < 0.001] was higher compared to 1-month [$\widehat{\mu }$ = 10.97] (Tables 5 and 6). Additionally, the average *positive PCQ physical* [$\widehat{\mu }$ = 5.81, $\widehat{\beta }$ = 2.43, *p* = 0.004], *social* [$\widehat{\mu }$ = 3.35, $\widehat{\beta }$ = 1.66, *p* = 0.008], and *total* [$\widehat{\mu }$ = 23.27, $\widehat{\beta }$ = 4.99, *p* < 0.001] scores at 5-years were higher compared to 1-month [*physical* $\widehat{\mu }$ = 3.38, *social* $\widehat{\mu }$ = 1.69, and *total* $\widehat{\mu }$ = 18.28] (Tables 5 and 6). There were no differences in the average *positive PCQ physical*, *social*, and *total* scores at 1-year compared to 1-month. These results were consistent with our MI analysis.

In our ACA, the average *positive PCQ physical* score decreased as age at baseline increases [$\widehat{\beta }$ = − 0.12, *p* = 0.044] (Table 6). Yet, this effect was not replicated in the MI analysis. Furthermore, the MI analysis estimated that the average *positive PCQ emotional* [${\widehat{\beta }}_{MI}$ = − 0.09, *p* = 0.028] and *total* [${\widehat{\beta }}_{MI}$ = − 0.15, *p* = 0.033] scores decreased as age at baseline increases. FPC participants had higher average *positive PCQ physical* [$\widehat{\beta }$ = 2.66, *p* = 0.028] and *social* [$\widehat{\beta }$ = 1.84, *p* = 0.034] scores, but these effects were not replicated in the MI analysis (Table 6). Participants that entered intensified screening had lower average within-participant *positive PCQ physical* [$\widehat{\beta }$ = − 4.31, *p* = 0.002] and *total* [$\widehat{\beta }$ = − 4.00, *p* = 0.024] scores compared to those who did not, which was consistent with our MI analysis (Table 6). There were no other covariates that were significantly associated with the *positive PCQ* scores, including EUS/MRI findings.

## Discussion

In our 5-year follow-up of individuals undergoing high-risk PDAC surveillance, we observed sustained participation and stable or improved psychological outcomes over time. Specifically, reductions in *IES intrusion* scores identified during early follow-up were maintained at the 5-year timepoint, including among participants with abnormal findings and those requiring intensified surveillance. These data provide important reassurance regarding the long-term acceptability and psychological safety of structured PDAC surveillance in high-risk individuals.

Variability in benefits and harms are reported in other cancer screening programs [27]. In population screening programs, transient increases in intrusion and avoidance measures of distress are typically below clinically-relevant levels, and often linked to abnormal or indeterminate findings, or among participants with higher perceived risk [28]. Perceived cancer risk and worry may influence engagement in research-based surveillance. However, high-risk individuals typically overestimate their cancer risk even after genetic counseling [29, 30]. Our participants appropriately perceived their *relative* cancer risk to be elevated, though we did not assess their perceived *absolute* risk. Whilst there was reasonable agreement on average between grouped PancPRO PDAC lifetime risk estimates and perceived relative PDAC chance in our FPC cohort, Nguyen et al. [30] found that participants’ subjective risks did not correlate to their PancPRO objective risk figures. Our baseline cancer worry and chance scores, supported by our baseline IES data, suggest a substantial proportion of participants had poor psychological function at enrolment, likely due to their lived experience. This supports Hart et al. [31]’s finding of clinically significant distress at baseline in one-quarter of their PDAC surveillance participants. For comparison, Bancroft et al. [32] showed that whilst *BRCA1* and *BRCA2* carriers undergoing prostate cancer screening reported significantly higher *IES intrusion* and *IES avoidance* scores compared to controls, their mean values remained below clinically relevant thresholds. Genetic counseling aims to promote empowerment to control health risks [33], and by engaging in PDAC surveillance, participants seek to control their cancer risk, which in turn supports psychological adaptation. Sustained psychological improvement in individuals who had undergone genetic counseling several years prior to enrolment suggests screening uptake may promote continued coping and adjustment to risk information.

Long-term adherence is a critical determinant of surveillance effectiveness, particularly given that PDAC risk is cumulative and surveillance is typically life-long. Overall, screening compliance in our cohort was high (88% prior to the COVID-19 pandemic), with most deferrals observed during the pandemic period and only eight participants choosing to withdraw. Compliance in PDAC screening protocols is currently poorly reported, though our compliance rate was higher than Al-Sukhni et al. [34] and Overbeek et al. [3] who reported 67% and 81%, respectively. Katona et al*.* [35] reported disruption to high-risk PDAC screening in the CAPS5 cohort with 97% of planned EUS procedures cancelled and 17% of patients deferring their procedure during COVID-19 pandemic restrictions. Our combined deferral rate of 53% during the pandemic likely reflects the stricter travel and healthcare restrictions in Sydney that were in place for essentially the entire 2-year pandemic period. The return to high compliance observed post-COVID-19 may reflect strong participant motivation and reassurance achieved through regular surveillance.

Importantly, our study suggests for the first time sustained psychological improvement after 5-years of continuous surveillance as measured by higher *positive PCQ* scores and lower *IES intrusion* scores. Overall, the number of participants reporting high *IES* distress scores was lower at each follow-up timepoint. The average *IES avoidance* and *IES total* scores decreased at 1-month and 1-year, and the average *IES intrusion* scores decreased at all follow-up timepoints relative to baseline. Together these results suggest IES scores decline over time after screening initiation. This observation was robust to adjustment for covariates in pre-specified models, and with sensitivity analyses accounting for missing data. These results do not show any evidence of harm in terms of IES from introduction of the intervention. Whilst there was a statistically significant increase in *IES avoidance* (all follow-up timepoints) *and IES intrusion* and *total IES* scores at 5-years in the low distress group, as measured by t-tests, this small increase is unlikely to be clinically meaningful, as the mean scores remained in the low range. Furthermore, this may have been influenced by other mental health impacts of COVID-19 restrictions at later timepoints. Whilst there was not a control group to infer a treatment effect, we suggest that the intervention may have reduced IES scores. However, we cannot formally infer a causal benefit from the intervention as such, as the reduction may have been due to regression to the mean over time.

Nonetheless, there was a significant age effect within our data. Younger participants reported higher cancer worry, perceived chance and *IES intrusion*, *IES avoidance* and *total IES* scores, indicating higher distress. Moreover, older participants reported lower negative impacts as measured by the *negative PCQ emotional, social* and *negative PCQ total* scores. This supports previous findings of higher distress in young patients undergoing PDAC surveillance [31, 36]. One-quarter of our cohort were aged ≤ 50 years at baseline, and almost half had at least one relative with young-onset PDAC (aged ≤ 50). Konings et al. [4] reported that the only factor associated with cancer worry was a relative with PDAC < 50 years (39% of their participants), which may also explain higher distress in our younger participants. Similar to our findings, Hart et al. [31] reported decreased *IES intrusion* scores at 3-months and 1-year follow-ups, with only younger participants showing reduced *IES avoidance* scores over time. Moreover, Anez-Bruzual et al. [37], reported significantly reduced distress at 4–6 weeks post-EUS, measured by a questionnaire containing *Health Belief Model* constructs. Both studies found that participants with poorer mental health and higher distress at baseline demonstrated the greatest improvement [31, 37]. Our categorized *IES* raw data, and ACA and MI analysis findings of higher *IES intrusion*, *IES avoidance* and *total IES* scores and higher *negative PCQ* scores in individuals with PHx depression support these earlier findings.

We detected a high proportion (53.8%) of abnormal screening findings, including three neoplastic lesions and progression of pre-malignant lesions in n = 28 (19.6%) participants. Cystic lesions (± duct communication) were identified in 32.9% of our participants, which is comparable to Lucas et al. [5] but lower than the 56% reported by Konings et al. [4]. Studies evaluating the psychological impact of colorectal and other abdominal cancer screening programs have reported higher *negative PCQ* scores following abnormal findings, false-positives or requirement for additional investigations [38, 39]. In the pancreatic setting, Marinelli et al. [40] reported increased stress, anxiety and lower health perception in non-high-risk individuals undergoing surveillance for IPMNs. However, this finding contrasts with Nieminen et al. [41] who found no difference in anxiety or health related quality of life for individuals undergoing IPMN surveillance. In our cohort, there were no significant differences in *IES*, *negative PCQ or positive PCQ* scores following abnormal EUS/MRI findings, supporting favorable psychological outcomes irrespective of screening findings. It is feasible that our result disclosure approach for screen-detected abnormalities, including multidisciplinary review for high-risk and concerning lesions, ensured adequate participant understanding, reassurance at the possibility of earlier detection, and mitigated psychological distress.

While most pancreatic cysts in the general population remain indolent [42], Overbeek et al. [8] reported that IPMNs in high-risk individuals showed higher growth rates and increased tendency to develop worrisome features or malignant progression, especially in pathogenic variant carriers. Reassuringly, Konings et al. [4] found no difference in cancer worry for those undergoing intensified surveillance or surgery. Overbeek et al. [9] reported a transient increase in cancer worry, but no change to anxiety and depression scores, in individuals requiring intensified surveillance. Moreover, despite considerable morbidity, individuals who underwent surgery felt it was justified and harbored favorable or unchanged opinions on their decision to proceed [9]. Importantly, in our study intensified surveillance was not associated with increased psychological distress. Participants undergoing more frequent investigation reported lower *positive PCQ physical* and *positive PCQ total* scores, without corresponding increases in *IES* or *negative PCQ* scores. *Positive PCQ physical* items included “been sleeping better”, “feeling more able to do things which you normally do” and “feeling more able to meet your home and work responsibilities”. There were no concomitant differences in *positive PCQ* scores for those with abnormal findings, suggesting that intensified surveillance may be associated with fewer ‘positive consequences’ or reduced improvement, potentially due to greater perceived physical or practical burdens, rather than adverse psychological impact. Differentiating logistical burden from psychological distress is critical when interpreting surveillance outcomes and may inform strategies to optimize patient experience without compromising clinical vigilance.

Overall, this study suggests that high-risk PDAC surveillance can be delivered with sustained compliance and without evidence of long-term psychological harm, as demonstrated by stable *negative PCQ total scores* from baseline to 5-years. Specifically, there were no increases in any of the *negative PCQ* subscales measuring the *emotional*, *physical* and *social* life domains. Indeed, the MI analysis suggested that there may have been a reduction in *negative PCQ total* score over the 5-years follow-up. Furthermore, there were no reported differences between those with normal or abnormal screening findings, or for those undergoing intensified surveillance.

### Clinical and research implications

These 5-year data extend our understanding of the psychological impact of repeated PDAC surveillance and suggest sustained improvement in psychological function and minimal harms, irrespective of clinical findings. Nonetheless, it highlights younger adults, individuals with depression and those requiring intensified investigation may benefit from additional support whilst engaged in PDAC surveillance. Inclusion of a genetic counselor in the screening program may explain our high compliance and can allow periodic assessment of coping and support mental health interventions when indicated. Screening coordinators and practitioners should be trained to assess for and respond to reports of cancer worry and have appropriate mechanisms in place to communicate abnormal findings to ensure they are accurately understood by the participants to minimize distress. Referral to mental health services, such as a psychologist should be available for those indicating prolonged distress.

Despite these reported psychological improvements, PDAC surveillance remains critically underutilized, with low uptake amongst eligible high-risk-individuals in international and Australian cohorts [15, 43, 44]. The views of these individuals are not captured in our current assessment of psychological impact. Additional research to address factors associated with non-uptake of PDAC surveillance may improve recognition of current barriers and support strategies to improve engagement by these individuals.

### Strengths and limitations

A strength of our study was the use of validated questionnaires to comprehensively assess the impacts of living with inherited cancer risk and ongoing cancer surveillance. The IES has been validated for use in assessing the psychological impact of inherited cancer risk [45] and the PCQ was specifically designed to measure the psychological consequences of cancer surveillance.

A well-documented limitation of studies evaluating PDAC screening in high-risk individuals, including the present study, is the absence of a control group [46, 47]. This reduces our ability to draw causal inferences as no counterfactual group is present for comparison. Accordingly, regression to mean may also explain changes to reported functioning overtime. Whilst our questionnaires asked participants to rate their feelings, thoughts and behaviors in relation to living with PDAC risk, we cannot exclude the possibility that their overall psychological function was influenced by other factors or stressors throughout the study period (e.g. the catastrophic 2019 Australian bushfires, and the COVID-19 pandemic). There is growing evidence of the varied psychological impacts of the COVID-19 pandemic, including increased anxiety, depression, sleep disturbance and maladaptive coping strategies (e.g. increased alcohol consumption and avoidance) both in Australia [48, 49] and internationally [50, 51]. It is feasible that the psychological impacts reported by some participants at 1-year and 5-years may have been influenced by poorer mental health during the COVID-19 pandemic, especially in the *IES avoidance* domain. Cancer worry and perceived risk were only assessed at baseline, so we are unable to directly assess if abnormal findings or intensified surveillance altered cancer worry or risk perception over time. Moreover, we acknowledge that our study design may have missed transient increases in distress following initial engagement with the screening program, of after abnormal screening findings, or intensified surveillance, especially between the 1-year to 5-years questionnaires. Additionally, as the number of participants undergoing multiple interval investigations was small, our data may not reflect the true impact of long-term intensified surveillance. Whilst the numbers were too small for a direct analysis, increased *IES* subscale and *total* scores and *negative PCQ* scores for some participants may have been linked to other external factors, such as a relative diagnosed with a pancreatic or non-pancreatic malignancy. Finally, as specific high-risk groups, such as *CDKN2A* pathogenic variant carriers, or individuals with PJS and Hereditary Pancreatitis were under-represented in our cohort, our findings may not be generalizable to all high-risk individuals.

There are also inherent biases within these data. Participants self-selected for study recruitment and likely had stronger motivations, greater cancer worry and/or higher perceived benefit(s) for involvement compared to those who chose not to proceed. Moreover, participants who perceived greater benefit(s) of participation may have been more likely to remain engaged, whereas those with lower perceived benefits more likely to withdraw by choice. As the study questionnaires were not completed following withdrawal, and some participants did not complete a questionnaire at all timepoints, these data may not reflect lower perceived benefits or negative impacts of a diagnosis.

## Conclusions

Despite being a resource-intensive process, accumulating evidence supports the possibility that high-risk PDAC surveillance is clinically beneficial, though long-term outcomes data are still required. Within the limitations of this observational study, we have addressed an important evidence-gap, with our data showing high screening compliance over 5-years and providing reassurance that prolonged participation does not confer cumulative psychological harm. These data are encouraging and support the long-term acceptability and psychological safety of continued surveillance in a structured, guideline-based surveillance program for appropriately selected and counseled high-risk individuals. Ongoing evaluation of psychological outcomes should remain an integral component of surveillance program assessment.

## Supplementary Information

Below is the link to the electronic supplementary material.

Supplementary Material 1. Baseline Questionnaire

Supplementary Material 2. 1-month Questionnaire

Supplementary Material 3. 1-year and 5-year Questionnaire

Supplementary Material 4. Table S1 and Table S2 Estimated Marginal Means on the latent scale, using generalized linear mixed model analyses

## Data Availability

The datasets generated and/or analyzed during the current study are available from the corresponding author on reasonable request.
