# Supplementary material for: Five-year psychological impact and surveillance compliance in the Australian Pancreatic Cancer Screening Program
Source: Fam Cancer. 2026 Apr 28;25(2):46. doi: 10.1007/s10689-026-00557-0 (PMC13124842; doi:10.1007/s10689-026-00557-0)
Supplement: Supplementary file 1 — Supplementary Material 1. Baseline Questionnaire [file 10689_2026_557_MOESM1_ESM.pdf]

St Vincent's Hospital
A facility of St Vincents & Mater Health Sydney

PANCREATIC CANCER SCREENING QUESTIONNAIRE

Please fill in the information requested. If you do not know how to answer a question, please leave it blank. We will contact you to complete any unanswered questions.

1. Last name: ______________________ First name: _____________________

2. Home address: ____
Street
____
City State Postcode

Daytime phone number: (____)_____________ Evening phone number: (____)________________

Email address: ___________________________ Mobile phone number: (____)________________

3. Name, address, and phone number of your primary doctor:

____
name
____
street address city state postcode

(___)_____________________
telephone

4. Your country of birth _____________________________

5. Your date of birth: ____/____/____ Current age: _____________ years

6. Current height: _________ cm

7. Current weight: _________kg Usual weight: _____________ kg

8. What is/was your usual occupation or job — the one worked at the longest?

Job/occupation ____Years in this job ____________

What did you do on this job? ____

In your work, was more time spent 1 ☐ indoors or 2 ☐ outdoors?

9. Your gender: ☐ male ☐ female

10. Your ethnic group (check one):

1 ☐ Caucasian 2 ☐ Asian 3 ☐ Aboriginal and Torres Island 4 ☐ African
5 ☐ Hispanic 6 ☐ Other ___________________________

11. a. Is your mother Jewish? 0 ☐ No 1 ☐ Yes
b. If yes, is she of Ashkenazi (Eastern European/Russian) background? 0 ☐ No 1 ☐ Yes
c. Is your father Jewish? 0 ☐ No 1 ☐ Yes
d. If yes, is he of Ashkenazi (Eastern European/Russian) background? 0 ☐ No 1 ☐ Yes

12. Marital status: 0 ☐ Single (never married) 1 ☐ Married 2 ☐ Separated
3 ☐ Divorced 4 ☐ Widowed

13. Have you ever had any cancers or tumors? 0 ☐ No 1 ☐ Yes

If yes, please describe what type of tumor, including your age when it was diagnosed.

			**<u>age at diagnosis</u>**
a. pancreas	0 ☐ No	1 ☐ Yes	____________ years
b. colon or rectal	0 ☐ No	1 ☐ Yes	____________ years
c. breast	0 ☐ No	1 ☐ Yes	____________ years
d. endometrial (uterus)	0 ☐ No	1 ☐ Yes	____________ years
e. cervix	0 ☐ No	1 ☐ Yes	____________ years
f. melanoma	0 ☐ No	1 ☐ Yes	____________ years
g. sarcoma	0 ☐ No	1 ☐ Yes	____________ years
h. brain	0 ☐ No	1 ☐ Yes	____________ years
i. stomach	0 ☐ No	1 ☐ Yes	____________ years
j. lung	0 ☐ No	1 ☐ Yes	____________ years
k. bile duct or gallbladder	0 ☐ No	1 ☐ Yes	____________ years
l. other ______________________________			____________ years

14. a. Has a doctor ever told you that you have or had sugar diabetes (diabetes mellitus)? 0 ☐ No 1 ☐ Yes

b. If yes, how old were you when you were first told that you had diabetes? _______ years

c. Do/did you take insulin? 0 ☐ No 1 ☐ Yes

d. Do/did you take any medications by mouth to control sugar? 0 ☐ No 1 ☐ Yes

15. a. Did a doctor ever tell you that you had pancreatitis? 0 ☐ No 1 ☐ Yes 2 ☐ Don't know

b. Did the doctor tell you what type of pancreatitis? 1 ☐ Acute 2 ☐ Chronic 3 ☐ Don't know

c. Did a doctor ever prescribe pancreatic enzymes for you? 0 ☐ No 1 ☐ Yes 2 ☐ Don't know

d. Did you ever have a pancreatic pseudocyst? 0 ☐ No 1 ☐ Yes 2 ☐ Don't know

e. Did you ever have gallstones? 0 ☐ No 1 ☐ Yes 2 ☐ Don't know

f. Have you ever had surgery on your belly? 0 ☐ No 1 ☐ Yes 2 ☐ Don't know

If yes, indicate the type of surgery (check all that apply):

1 ☐ gallbladder removal (cholecystectomy) 2 ☐ appendix removal (appendectomy)
3 ☐ gastric surgery 4 ☐ surgery on small or large intestines
5 ☐ other ____

g. Have you ever had: celiac disease: 0 ☐ No 1 ☐ Yes 2 ☐ Don't know
sclerosing cholangitis (PSC): 0 ☐ No 1 ☐ Yes 2 ☐ Don't know
gluten sensitive enteropathy: 0 ☐ No 1 ☐ Yes 2 ☐ Don't know

h. Do you have any medical problems? 1 ☐ No 1 ☐ Yes

If yes, what are they?

- 1 ☐ hypertension
- 2 ☐ asthma
- 3 ☐ congestive heart failure
- 4 ☐ coronary artery disease (heart attack, angina)
- 5 ☐ peripheral vascular disease
- 6 ☐ stroke
- 7 ☐ connective tissue disease
- 8 ☐ peptic ulcer disease
- 9 ☐ mild liver disease
- 10 ☐ chronic pulmonary disease (COPD)
- 11 ☐ paralysis of one side of the body
- 12 ☐ moderate or severe kidney disease
- 13 ☐ any tumor (specify):______________
- 14 ☐ diabetes with kidney, eye, or nerve complications
- 15 ☐ leukemia
- 16 ☐ lymphoma
- 17 ☐ moderate or severe liver disease
- 18 ☐ metastatic cancer (specify):_______________
- 19 ☐ AIDS
- 20 ☐ depression, requiring medication
- 21 ☐ sleep apnea
- 22 ☐ other (specify):_________________________

Do you have any history of:

i. Colon polyps — 0 ☐ No 1 ☐ Yes 2 ☐ Don't know
j. Barrett's esophagus — 0 ☐ No 1 ☐ Yes 2 ☐ Don't know
k. Acid reflux disease or recurrent heartburn/regurgitation — 0 ☐ No 1 ☐ Yes 2 ☐ Don't know
l. Colitis — 0 ☐ No 1 ☐ Yes 2 ☐ Don't know

m. Have you ever had diarrhoea or loose stools for more than 6 weeks? 0 ☐ No 1 ☐ Yes

n. Do you currently have pain or discomfort in the upper part of your belly? 0 ☐ No 1 ☐ Yes

o. Do you currently have a poor appetite? 0 ☐ No 1 ☐ Yes

p. Have you lost weight in the last 6 months? 0 ☐ No 1 ☐ Yes

q. If yes, how much weight have you lost?
1 ☐ less than 5kg 2 ☐ 5-10kg 3 ☐ more than 10kg

r. Have you ever had darkening of your urine (tea-color) or yellowing of your eyes?
0 ☐ No 1 ☐ Yes

s. Do you have any other symptoms that are bothering you? 0 ☐ No 1 ☐ Yes (specify):
____
____

t. Do you have any allergies: 0 ☐ No 1 ☐ Yes
If yes, specify: ____

u. Have you ever had any problems with sedation for medical procedures or anesthesia for surgery?

0 ☐ No 1 ☐ Yes
If yes, specify: ____

16. Does your spouse have a history of pancreas cancer? 0 ☐ No 1 ☐ Yes 2 ☐ Don't know
If yes, please explain on back of form.

17. Have you ever been told that you have:
a. Peutz-Jeghers Syndrome — 0 ☐ No 1 ☐ Yes
b. Familial Atypical Multiple Mole and Melanoma (FAMMM) Syndrome — 0 ☐ No 1 ☐ Yes
c. Familial Breast Cancer — 0 ☐ No 1 ☐ Yes
d. Familial Pancreatitis — 0 ☐ No 1 ☐ Yes
e. Hereditary Non-Polyposis Colon Cancer (HNPCC) Syndrome — 0 ☐ No 1 ☐ Yes

18. a. Do you ever smoke cigarettes now? 0 ☐ No 1 ☐ Yes

b. Did you ever smoke regularly (> 100 cigarettes in lifetime) 0 ☐ No 1 ☐ Yes

For Current and Former Smokers Only:

a. How many cigarettes do/did you smoke per day on average? _________

b. Was there ever a time when you regularly smoked more cigarettes per day than you do now?
0 ☐ No 1 ☐ Yes, I used to smoke _______ cigarettes per day

c. How many years have you been smoking? __________

d. Since the time you began smoking, what was the longest period of time you have gone without smoking any cigarettes at all? _________

e. When you were smoking, how many cigarettes did you usually smoke?
_________ (choose one) 1 ☐ per day
2 ☐ per week
3 ☐ per month

f. How many years did you smoke? ___________

g. How long has it been since you stopped smoking?
_________ (choose one) 1 ☐ days
2 ☐ weeks
3 ☐ months
4 ☐ years

h. What were your reasons for quitting smoking?

____

____

	Never	Occasionally	Current Regularly	Past	
Do you smoke pipes or cigars?	☐	☐	☐	☐	How long? _____ years
Do you use chewing tobacco?	☐	☐	☐	☐	How long? _____ years
Do you use snuff?	☐	☐	☐	☐	How long? _____ years

19. Did you **regularly spend 1 hour or more per day** in a room (at home or work) where someone (else) was smoking? Place a check mark in boxes if true:

**When you were:**	**NO**	**YES**			
		**Parent**	**Spouse**	**Co-worker**	**Other (specify relationship)**
0 - 10 years old?					
11 - 20 years old?					
21 - 30 years old?					
31 - 40 years old?					

41 - 50 years old?					
51 - 60 years old?					
Older than 60 years old?					

20. How many individuals in your immediate household smoke (not including yourself)? __________

21. a. Have you ever consumed any alcoholic beverages on a regular basis? 0 ☐ No 1 ☐ Yes
b. At what age did you start drinking alcoholic drinks? ____________ years
c. Are you still drinking alcoholic beverages on a regular basis? 0 ☐ No 1 ☐ Yes
d. If you have stopped drinking alcoholic beverages, at what age did you stop? ____________ years

**BEVERAGE**	**HOW OFTEN**						
	Less than 1 per week	2 - 4 per week	5 – 6 per week	1 per day	2 - 3 per day	4 – 5 per day	6+ per day
Beer							
Wine							
Spirits (whiskey, gin, etc)							

22. What is your usual serving size of alcohol?

☐ 1 can/bottle of beer or 1 glass of wine or 1 shot

☐ 2 cans/bottles of beer or glasses of wine or shots

☐ 3 or more cans/bottles of beer, glasses of wine or shots.

23. a. Do you take aspirin or aspirin-containing medications on a regular basis (at least 3 times per week for > 4 weeks) ?
0 ☐ No 1 ☐ Yes

b. How long have you been taking this medication? 1 ☐ < 1 year 2 ☐ 1-5 years 3 ☐ > 5 years

24. a. Do you take non-steroidal anti-inflammatory drugs (NSAIDS) on a regular basis? 0 ☐ No 1 ☐ Yes

b. Which NSAID(s) are you taking?

☐ ibuprofen (Nurofen ,Advil,Bugesic) ☐ anaprox (Naproxen) ☐ diclofenac (Voltaren,Dinac)
☐ indomethacin (Indocid,Arthrexin) ☐ other_______________________________

c. How long have you been taking NSAIDs? 1 ☐ < 1 year 2 ☐ 1-5 years 3 ☐ > 5 years

25. a. Do/did you take celecoxib (Celebrex) or Mobic on a regular basis? 0 ☐ No 1 ☐ Yes

b. How long have you been taking this medication? 1 ☐ < 1 year 2 ☐ 1-5 years 3 ☐ > 5 years

26. a. Do you take any medication that contains folate (folic acid)? 0 ☐ No 1 ☐ Yes

b. How long have you been taking this medication? ☐ < 1 year ☐ 1-5 years ☐ > 5 years

27. What medications not on the above list did you take for more than six months?

Medication Name	Reason for taking this medication	Amount per dose	Doses per day	How long have you taken this medication?

28. a. Have you ever been treated for a mental health problem? 0 ☐ No 1 ☐ Yes

If yes, please describe____

b. In general, how would you rate your mood during the past 12 months?

Extremely bad — Extremely good

1 2 3 4 5 6 7 8 9 10

29. How do you feel about your life now? (Place an X mark in the corresponding box)

☐ 1) Delighted
☐ 2) Pleased
☐ 3) Mostly satisfied
☐ 4) Mixed (about equally satisfied and dissatisfied)
☐ 5) Mostly dissatisfied
☐ 6) Unhappy
☐ 7) Terrible

30. We have asked a little about various parts of your life. Now we want to ask about your life as a whole. How satisfied are you with your life as a whole these days?

Circle the single number which comes closes to how satisfied or dissatisfied you are with your life as a whole.

**Completely satisfied**	1	2	3	4	5	6	7	**Completely dissatisfied**

31. How worried are you that you may develop pancreatic cancer? (Choose one below)

Not at all worried — Average — Extremely worried

1 ☐ 2 ☐ 3 ☐ 4 ☐ 5 ☐

b. How worried are you that you may develop another type of cancer? (Choose one below)

Not at all worried — Average — Extremely worried

1 ☐ 2 ☐ 3 ☐ 4 ☐ 5 ☐

c. Compared to most people of your same age, sex, and race, what do you think your chances are of getting pancreatic cancer sometime in your lifetime? (Choose one below)

Much below others				Much above others
1 ☐	2 ☐	3 ☐	4 ☐	5 ☐

d. Compared to most people of your same age, sex, and race, what do you think your chances are of getting another type of cancer sometime in your lifetime? (Choose one below)

Much below others				Much above others
1 ☐	2 ☐	3 ☐	4 ☐	5 ☐

32. **IMPACT OF EVENTS SCALE**

Below is a list of comments made by people about being at risk for pancreatic cancer. Please tick a box to indicate how frequently these comments were true for you during *the last seven days*

		Not at all	Rarely	Sometimes	Often
a.	I thought about it when I didn't mean to	❑	❑	❑	❑
b.	I avoided letting myself get upset when I thought about or was reminded of it	❑	❑	❑	❑
c.	I tried to remove it from my memory	❑	❑	❑	❑
d.	I had trouble falling asleep or staying asleep because of pictures or thoughts about it that came into my mind	❑	❑	❑	❑
e.	I had waves of strong feelings about it	❑	❑	❑	❑
f.	I had dreams about it	❑	❑	❑	❑
g.	I stayed away from reminders of it	❑	❑	❑	❑
h.	I felt as if it wasn't real	❑	❑	❑	❑
i.	I tried not to talk about it	❑	❑	❑	❑
j.	Pictures popped up into my mind	❑	❑	❑	❑
k.	Other things kept making me think about it	❑	❑	❑	❑
l.	I was aware that I still had a lot of feelings about it, but I didn't deal with them	❑	❑	❑	❑
m.	I tried not to think about it	❑	❑	❑	❑
n.	Any reminder brought back feelings about it	❑	❑	❑	❑
o.	My feelings were sort of numb	❑	❑	❑	❑

33. **Personal Consequences Questionnaire**

We would like to know your thoughts and feelings about pancreatic cancer.

Over the ***last week*** have you experienced the following things because of thoughts and feelings about *pancreatic cancer*?

	Not at all	Rarely	Some of the time	Quite a lot of the time
a. Had trouble sleeping	0	1	2	3
b. Experienced a change in appetite	0	1	2	3
c. Been unhappy or depressed	0	1	2	3
d. Been scared and panicky	0	1	2	3
e. Felt nervous or strung up	0	1	2	3
f. Felt under strain	0	1	2	3
g. Found you have been keeping things from those who are close to you	0	1	2	3
h. Found yourself taking things out on other people	0	1	2	3
i. Found yourself noticeable withdrawing from those who are close to you	0	1	2	3
j. Had difficulty doing things around the house which you normally do	0	1	2	3
k. Had difficulty meeting work or other commitments	0	1	2	3
l. Feeling worried about your future	0	1	2	3

PLEASE FILL IN YOUR FAMILY HISTORY

Please fill in the table. This is very important!

YOUR PARENTS AND GRANDPARENTS	DATE OF BIRTH Day/Mth/Yr	ANY PANCREAS PROBLEMS?	ANY OTHER CANCERS?	SMOKER? Smoker = >100 cigarettes in lifetime	DECEASED?
Your mother		**Pancreas Cancer:** ☐ Yes ☐ No ☐Unknown<br>**Diabetes:** ☐ Yes ☐ No ☐Unknown<br>**Pancreatitis**: ☐ Yes ☐ No ☐Unknown<br><br>Age(s) at Diagnosis:	☐ Yes ☐ No<br>☐ Unknown<br>Type:<br><br>Age at Diagnosis:	☐ Never<br>☐ Occasional<br>☐ Regular<br>☐ Unknown<br># Years Smoked:<br># Cigarettes/Day:	☐Yes ☐ No<br><br>Age At Death:<br>Cause of Death:
Your father		**Pancreas Cancer:** ☐ Yes ☐ No ☐Unknown<br>**Diabetes:** ☐ Yes ☐ No ☐Unknown<br>**Pancreatitis**: ☐ Yes ☐ No ☐Unknown<br><br>Age(s) at Diagnosis:	☐ Yes ☐ No<br>☐ Unknown<br>Type:<br><br>Age at Diagnosis:	☐ Never<br>☐ Occasional<br>☐ Regular<br>☐ Unknown<br># Years Smoked:<br># Cigarettes/Day:	☐Yes ☐ No<br><br>Age At Death:<br>Cause of Death:
Your mother's mother		**Pancreas Cancer:** ☐ Yes ☐ No ☐Unknown<br>**Diabetes:** ☐ Yes ☐ No ☐Unknown<br>**Pancreatitis**: ☐ Yes ☐ No ☐Unknown<br><br>Age(s) at Diagnosis:	☐ Yes ☐ No<br>☐ Unknown<br>Type:<br><br>Age at Diagnosis:	☐ Never<br>☐ Occasional<br>☐ Regular<br>☐ Unknown<br># Years Smoked:<br># Cigarettes/Day:	☐Yes ☐ No<br><br>Age At Death:<br>Cause of Death:
Your mother's father		**Pancreas Cancer:** ☐ Yes ☐ No ☐Unknown<br>**Diabetes:** ☐ Yes ☐ No ☐Unknown<br>**Pancreatitis**: ☐ Yes ☐ No ☐Unknown<br><br>Age(s) at Diagnosis:	☐ Yes ☐ No<br>☐ Unknown<br>Type:<br><br>Age at Diagnosis:	☐ Never<br>☐ Occasional<br>☐ Regular<br>☐ Unknown<br># Years Smoked:<br># Cigarettes/Day:	☐Yes ☐ No<br><br>Age At Death:<br>Cause of Death:
Your father's mother		**Pancreas Cancer:** ☐ Yes ☐ No ☐Unknown<br>**Diabetes:** ☐ Yes ☐ No ☐Unknown<br>**Pancreatitis**: ☐ Yes ☐ No ☐Unknown<br><br>Age(s) at Diagnosis:	☐ Yes ☐ No<br>☐ Unknown<br>Type:<br><br>Age at Diagnosis:	☐ Never<br>☐ Occasional<br>☐ Regular<br>☐ Unknown<br># Years Smoked:<br># Cigarettes/Day:	☐Yes ☐ No<br><br>Age At Death:<br>Cause of Death:
Your father's father		**Pancreas Cancer:** ☐ Yes ☐ No ☐Unknown<br>**Diabetes:** ☐ Yes ☐ No ☐Unknown<br>**Pancreatitis**: ☐ Yes ☐ No ☐Unknown<br><br>Age(s) at Diagnosis:	☐ Yes ☐ No<br>☐ Unknown<br>Type:<br><br>Age at Diagnosis:	☐ Never<br>☐ Occasional<br>☐ Regular<br>☐ Unknown<br># Years Smoked:<br># Cigarettes/Day:	☐Yes ☐ No<br><br>Age At Death:<br>Cause of Death:

St Vincent's Hospital

How many brothers do/did you have? _______ How many sisters do/did you have? _______ (Use additional sheets as needed)

* If any of your brothers and sisters have different parents, please indicate which are half-sibs and whether blood relationship is through patient's mother or patient's father.

**YOUR BROTHERS AND SISTERS**	**DATE OF BIRTH Day/Mth/Yr**	**ANY PANCREAS PROBLEMS?**	**ANY OTHER CANCERS?**	**SMOKER?** Smoker = >100 cigarettes in Lifetime	**DECEASED?**
Brother #1		**Pancreas Cancer:** ☐ Yes ☐ No ☐Unknown<br>**Diabetes:** ☐ Yes ☐ No ☐Unknown<br>**Pancreatitis**: ☐ Yes ☐ No ☐Unknown<br>Age(s) at Diagnosis:	☐ Yes ☐ No<br>☐ Unknown<br>Type:<br>Age at Diagnosis:	☐ Never<br>☐ Occasional<br>☐ Regular<br>☐ Unknown<br># Years Smoked:<br># Cigarettes/Day:	☐Yes ☐ No<br>Age At Death:<br>Cause of Death:
Brother #2		**Pancreas Cancer:** ☐ Yes ☐ No ☐Unknown<br>**Diabetes:** ☐ Yes ☐ No ☐Unknown<br>**Pancreatitis**: ☐ Yes ☐ No ☐Unknown<br>Age(s) at Diagnosis:	☐ Yes ☐ No<br>☐ Unknown<br>Type:<br>Age at Diagnosis:	☐ Never<br>☐ Occasional<br>☐ Regular<br>☐ Unknown<br># Years Smoked:<br># Cigarettes/Day:	☐Yes ☐ No<br>Age At Death:<br>Cause of Death:
Brother #3		**Pancreas Cancer:** ☐ Yes ☐ No ☐Unknown<br>**Diabetes:** ☐ Yes ☐ No ☐Unknown<br>**Pancreatitis**: ☐ Yes ☐ No ☐Unknown<br>Age(s) at Diagnosis:	☐ Yes ☐ No<br>☐ Unknown<br>Type:<br>Age at Diagnosis:	☐ Never<br>☐ Occasional<br>☐ Regular<br>☐ Unknown<br># Years Smoked:<br># Cigarettes/Day:	☐Yes ☐ No<br>Age At Death:<br>Cause of Death:
Sister #1		**Pancreas Cancer:** ☐ Yes ☐ No ☐Unknown<br>**Diabetes:** ☐ Yes ☐ No ☐Unknown<br>**Pancreatitis**: ☐ Yes ☐ No ☐Unknown<br>Age(s) at Diagnosis:	☐ Yes ☐ No<br>☐ Unknown<br>Type:<br>Age at Diagnosis:	☐ Never<br>☐ Occasional<br>☐ Regular<br>☐ Unknown<br># Years Smoked:<br># Cigarettes/Day:	☐Yes ☐ No<br>Age At Death:<br>Cause of Death:
Sister #2		**Pancreas Cancer:** ☐ Yes ☐ No ☐Unknown<br>**Diabetes:** ☐ Yes ☐ No ☐Unknown<br>**Pancreatitis**: ☐ Yes ☐ No ☐Unknown<br>Age(s) at Diagnosis:	☐ Yes ☐ No<br>☐ Unknown<br>Type:<br>Age at Diagnosis:	☐ Never<br>☐ Occasional<br>☐ Regular<br>☐ Unknown<br># Years Smoked:<br># Cigarettes/Day:	☐Yes ☐ No<br>Age At Death:<br>Cause of Death:
Sister #3		**Pancreas Cancer:** ☐ Yes ☐ No ☐Unknown<br>**Diabetes:** ☐ Yes ☐ No ☐Unknown<br>**Pancreatitis**: ☐ Yes ☐ No ☐Unknown<br>Age(s) at Diagnosis:	☐ Yes ☐ No<br>☐ Unknown<br>Type:<br>Age at Diagnosis:	☐ Never<br>☐ Occasional<br>☐ Regular<br>☐ Unknown<br># Years Smoked:<br># Cigarettes/Day:	☐Yes ☐ No<br>Age At Death:<br>Cause of Death:

St Vincent's Hospital

How many sons do/did you have? _______ How many daughters do/did you have? _______

Please complete the following information on your children. (Use additional sheets as needed)

YOUR CHILDREN	DATE OF BIRTH Day/Mth/Yr	ANY PANCREAS PROBLEMS?	ANY OTHER CANCERS?	SMOKER? Smoker = >100 cigarettes in lifetime	DECEASED?
Son #1		**Pancreas Cancer:** ☐ Yes ☐ No ☐Unknown<br>**Diabetes:** ☐ Yes ☐ No ☐Unknown<br>**Pancreatitis**: ☐ Yes ☐ No ☐Unknown<br>Age(s) at Diagnosis:	☐ Yes ☐ No<br>☐ Unknown<br>Type:<br>Age at Diagnosis:	☐ Never<br>☐ Occasional<br>☐ Regular<br>☐ Unknown<br># Years Smoked:<br># Cigarettes/Day:	☐Yes ☐ No<br>Age At Death:<br>Cause of Death:
Son #2		**Pancreas Cancer:** ☐ Yes ☐ No ☐Unknown<br>**Diabetes:** ☐ Yes ☐ No ☐Unknown<br>**Pancreatitis**: ☐ Yes ☐ No ☐Unknown<br>Age(s) at Diagnosis:	☐ Yes ☐ No<br>☐ Unknown<br>Type:<br>Age at Diagnosis:	☐ Never<br>☐ Occasional<br>☐ Regular<br>☐ Unknown<br># Years Smoked:<br># Cigarettes/Day:	☐Yes ☐ No<br>Age At Death:<br>Cause of Death:
Son #3		**Pancreas Cancer:** ☐ Yes ☐ No ☐Unknown<br>**Diabetes:** ☐ Yes ☐ No ☐Unknown<br>**Pancreatitis**: ☐ Yes ☐ No ☐Unknown<br>Age(s) at Diagnosis:	☐ Yes ☐ No<br>☐ Unknown<br>Type:<br>Age at Diagnosis:	☐ Never<br>☐ Occasional<br>☐ Regular<br>☐ Unknown<br># Years Smoked:<br># Cigarettes/Day:	☐Yes ☐ No<br>Age At Death:<br>Cause of Death:
Daughter #1		**Pancreas Cancer:** ☐ Yes ☐ No ☐Unknown<br>**Diabetes:** ☐ Yes ☐ No ☐Unknown<br>**Pancreatitis**: ☐ Yes ☐ No ☐Unknown<br>Age(s) at Diagnosis:	☐ Yes ☐ No<br>☐ Unknown<br>Type:<br>Age at Diagnosis:	☐ Never<br>☐ Occasional<br>☐ Regular<br>☐ Unknown<br># Years Smoked:<br># Cigarettes/Day:	☐Yes ☐ No<br>Age At Death:<br>Cause of Death:
Daughter #2		**Pancreas Cancer:** ☐ Yes ☐ No ☐Unknown<br>**Diabetes:** ☐ Yes ☐ No ☐Unknown<br>**Pancreatitis**: ☐ Yes ☐ No ☐Unknown<br>Age(s) at Diagnosis:	☐ Yes ☐ No<br>☐ Unknown<br>Type:<br>Age at Diagnosis:	☐ Never<br>☐ Occasional<br>☐ Regular<br>☐ Unknown<br># Years Smoked:<br># Cigarettes/Day:	☐Yes ☐ No<br>Age At Death:<br>Cause of Death:
Daughter #3		**Pancreas Cancer:** ☐ Yes ☐ No ☐Unknown<br>**Diabetes:** ☐ Yes ☐ No ☐Unknown<br>**Pancreatitis**: ☐ Yes ☐ No ☐Unknown<br>Age(s) at Diagnosis:	☐ Yes ☐ No<br>☐ Unknown<br>Type:<br>Age at Diagnosis:	☐ Never<br>☐ Occasional<br>☐ Regular<br>☐ Unknown<br># Years Smoked:<br># Cigarettes/Day:	☐Yes ☐ No<br>Age At Death:<br>Cause of Death:

Please complete for your <u>mother's</u> brothers and sisters. (Use additional sheets as needed)

UNCLES AND AUNTS	DATE OF BIRTH Day/Mth/Yr	ANY PANCREAS PROBLEMS?	ANY OTHER CANCERS?	SMOKER? Smoker = >100 cigarettes in lifetime	DECEASED?
Mother's brother #1		**Pancreas Cancer:** ☐ Yes ☐ No ☐Unknown<br>**Diabetes:** ☐ Yes ☐ No ☐Unknown<br>**Pancreatitis**: ☐ Yes ☐ No ☐Unknown<br><br>Age(s) at Diagnosis:	☐ Yes ☐ No<br>☐ Unknown<br>Type:<br><br>Age at Diagnosis:	☐ Never<br>☐ Occasional<br>☐ Regular<br>☐ Unknown<br># Years Smoked:<br># Cigarettes/Day:	☐Yes ☐ No<br><br>Age At Death:<br>Cause of Death:
Mother's brother #2		**Pancreas Cancer:** ☐ Yes ☐ No ☐Unknown<br>**Diabetes:** ☐ Yes ☐ No ☐Unknown<br>**Pancreatitis**: ☐ Yes ☐ No ☐Unknown<br><br>Age(s) at Diagnosis:	☐ Yes ☐ No<br>☐ Unknown<br>Type:<br><br>Age at Diagnosis:	☐ Never<br>☐ Occasional<br>☐ Regular<br>☐ Unknown<br># Years Smoked:<br># Cigarettes/Day:	☐Yes ☐ No<br><br>Age At Death:<br>Cause of Death:
Mother's brother #3		**Pancreas Cancer:** ☐ Yes ☐ No ☐Unknown<br>**Diabetes:** ☐ Yes ☐ No ☐Unknown<br>**Pancreatitis**: ☐ Yes ☐ No ☐Unknown<br><br>Age(s) at Diagnosis:	☐ Yes ☐ No<br>☐ Unknown<br>Type:<br><br>Age at Diagnosis:	☐ Never<br>☐ Occasional<br>☐ Regular<br>☐ Unknown<br># Years Smoked:<br># Cigarettes/Day:	☐Yes ☐ No<br><br>Age At Death:<br>Cause of Death:
Mother's sister #1		**Pancreas Cancer:** ☐ Yes ☐ No ☐Unknown<br>**Diabetes:** ☐ Yes ☐ No ☐Unknown<br>**Pancreatitis**: ☐ Yes ☐ No ☐Unknown<br><br>Age(s) at Diagnosis:	☐ Yes ☐ No<br>☐ Unknown<br>Type:<br><br>Age at Diagnosis:	☐ Never<br>☐ Occasional<br>☐ Regular<br>☐ Unknown<br># Years Smoked:<br># Cigarettes/Day:	☐Yes ☐ No<br><br>Age At Death:<br>Cause of Death:
Mother's sister #2		**Pancreas Cancer:** ☐ Yes ☐ No ☐Unknown<br>**Diabetes:** ☐ Yes ☐ No ☐Unknown<br>**Pancreatitis**: ☐ Yes ☐ No ☐Unknown<br><br>Age(s) at Diagnosis:	☐ Yes ☐ No<br>☐ Unknown<br>Type:<br><br>Age at Diagnosis:	☐ Never<br>☐ Occasional<br>☐ Regular<br>☐ Unknown<br># Years Smoked:<br># Cigarettes/Day:	☐Yes ☐ No<br><br>Age At Death:<br>Cause of Death:
Mother's sister #3		**Pancreas Cancer:** ☐ Yes ☐ No ☐Unknown<br>**Diabetes:** ☐ Yes ☐ No ☐Unknown<br>**Pancreatitis**: ☐ Yes ☐ No ☐Unknown<br><br>Age(s) at Diagnosis:	☐ Yes ☐ No<br>☐ Unknown<br>Type:<br><br>Age at Diagnosis:	☐ Never<br>☐ Occasional<br>☐ Regular<br>☐ Unknown<br># Years Smoked:<br># Cigarettes/Day:	☐Yes ☐ No<br><br>Age At Death:<br>Cause of Death:

Please complete for your father's brothers and sisters. (Use additional sheets as needed)

UNCLES & AUNTS	DATE OF BIRTH Day/Mth/Yr	ANY PANCREAS PROBLEMS?	ANY OTHER CANCERS?	SMOKER? Smoker = >100 cigarettes in lifetime	DECEASED?
Father's brother #1		**Pancreas Cancer:** ☐ Yes ☐ No ☐Unknown<br>**Diabetes:** ☐ Yes ☐ No ☐Unknown<br>**Pancreatitis**: ☐ Yes ☐ No ☐Unknown<br><br>Age(s) at Diagnosis:	☐ Yes ☐ No<br>☐ Unknown<br>Type:<br><br>Age at Diagnosis:	☐ Never<br>☐ Occasional<br>☐ Regular<br>☐ Unknown<br># Years Smoked:<br># Cigarettes/Day:	☐Yes ☐ No<br><br>Age At Death:<br>Cause of Death:
Father's brother #2		**Pancreas Cancer:** ☐ Yes ☐ No ☐Unknown<br>**Diabetes:** ☐ Yes ☐ No ☐Unknown<br>**Pancreatitis**: ☐ Yes ☐ No ☐Unknown<br><br>Age(s) at Diagnosis:	☐ Yes ☐ No<br>☐ Unknown<br>Type:<br><br>Age at Diagnosis:	☐ Never<br>☐ Occasional<br>☐ Regular<br>☐ Unknown<br># Years Smoked:<br># Cigarettes/Day:	☐Yes ☐ No<br><br>Age At Death:<br>Cause of Death:
Father's brother #3		**Pancreas Cancer:** ☐ Yes ☐ No ☐Unknown<br>**Diabetes:** ☐ Yes ☐ No ☐Unknown<br>**Pancreatitis**: ☐ Yes ☐ No ☐Unknown<br><br>Age(s) at Diagnosis:	☐ Yes ☐ No<br>☐ Unknown<br>Type:<br><br>Age at Diagnosis:	☐ Never<br>☐ Occasional<br>☐ Regular<br>☐ Unknown<br># Years Smoked:<br># Cigarettes/Day:	☐Yes ☐ No<br><br>Age At Death:<br>Cause of Death:
Father's sister #1		**Pancreas Cancer:** ☐ Yes ☐ No ☐Unknown<br>**Diabetes:** ☐ Yes ☐ No ☐Unknown<br>**Pancreatitis**: ☐ Yes ☐ No ☐Unknown<br><br>Age(s) at Diagnosis:	☐ Yes ☐ No<br>☐ Unknown<br>Type:<br><br>Age at Diagnosis:	☐ Never<br>☐ Occasional<br>☐ Regular<br>☐ Unknown<br># Years Smoked:<br># Cigarettes/Day:	☐Yes ☐ No<br><br>Age At Death:<br>Cause of Death:
Father's sister #2		**Pancreas Cancer:** ☐ Yes ☐ No ☐Unknown<br>**Diabetes:** ☐ Yes ☐ No ☐Unknown<br>**Pancreatitis**: ☐ Yes ☐ No ☐Unknown<br><br>Age(s) at Diagnosis:	☐ Yes ☐ No<br>☐ Unknown<br>Type:<br><br>Age at Diagnosis:	☐ Never<br>☐ Occasional<br>☐ Regular<br>☐ Unknown<br># Years Smoked:<br># Cigarettes/Day:	☐Yes ☐ No<br><br>Age At Death:<br>Cause of Death:
Father's sister #3		**Pancreas Cancer:** ☐ Yes ☐ No ☐Unknown<br>**Diabetes:** ☐ Yes ☐ No ☐Unknown<br>**Pancreatitis**: ☐ Yes ☐ No ☐Unknown<br><br>Age(s) at Diagnosis:	☐ Yes ☐ No<br>☐ Unknown<br>Type:<br><br>Age at Diagnosis:	☐ Never<br>☐ Occasional<br>☐ Regular<br>☐ Unknown<br># Years Smoked:<br># Cigarettes/Day:	☐Yes ☐ No<br><br>Age At Death:<br>Cause of Death:

Do you have any other blood relatives (alive or deceased) who have been diagnosed with pancreas cancer or other cancer? (not listed in questions 35-39) **0** ☐ **No** **1** ☐ **Yes** **If yes, please fill in the table below.**

**RELATIONSHIP TO YOU**	**DATE OF BIRTH Day/Mth/Yr**	**ANY PANCREAS PROBLEMS?**	**ANY OTHER CANCERS?**	**SMOKER?**<br>Smoker = >100 cigarettes in lifetime	**DECEASED?**
		**Pancreas Cancer:** ☐ Yes ☐ No ☐Unknown<br>**Diabetes:** ☐ Yes ☐ No ☐Unknown<br>**Pancreatitis**: ☐ Yes ☐ No ☐Unknown<br>Age(s) at Diagnosis:	☐ Yes ☐ No<br>☐ Unknown<br>Type:<br>Age at Diagnosis:	☐ Never<br>☐ Occasional<br>☐ Regular<br>☐ Unknown<br># Years Smoked:<br># Cigarettes/Day:	☐Yes ☐ No<br>Age At Death:<br>Cause of Death:
		**Pancreas Cancer:** ☐ Yes ☐ No ☐Unknown<br>**Diabetes:** ☐ Yes ☐ No ☐Unknown<br>**Pancreatitis**: ☐ Yes ☐ No ☐Unknown<br>Age(s) at Diagnosis:	☐ Yes ☐ No<br>☐ Unknown<br>Type:<br>Age at Diagnosis:	☐ Never<br>☐ Occasional<br>☐ Regular<br>☐ Unknown<br># Years Smoked:<br># Cigarettes/Day:	☐Yes ☐ No<br>Age At Death:<br>Cause of Death:
		**Pancreas Cancer:** ☐ Yes ☐ No ☐Unknown<br>**Diabetes:** ☐ Yes ☐ No ☐Unknown<br>**Pancreatitis**: ☐ Yes ☐ No ☐Unknown<br>Age(s) at Diagnosis:	☐ Yes ☐ No<br>☐ Unknown<br>Type:<br>Age at Diagnosis:	☐ Never<br>☐ Occasional<br>☐ Regular<br>☐ Unknown<br># Years Smoked:<br># Cigarettes/Day:	☐Yes ☐ No<br>Age At Death:<br>Cause of Death:
		**Pancreas Cancer:** ☐ Yes ☐ No ☐Unknown<br>**Diabetes:** ☐ Yes ☐ No ☐Unknown<br>**Pancreatitis**: ☐ Yes ☐ No ☐Unknown<br>Age(s) at Diagnosis:	☐ Yes ☐ No<br>☐ Unknown<br>Type:<br>Age at Diagnosis:	☐ Never<br>☐ Occasional<br>☐ Regular<br>☐ Unknown<br># Years Smoked:<br># Cigarettes/Day:	☐Yes ☐ No<br>Age At Death:<br>Cause of Death:
		**Pancreas Cancer:** ☐ Yes ☐ No ☐Unknown<br>**Diabetes:** ☐ Yes ☐ No ☐Unknown<br>**Pancreatitis**: ☐ Yes ☐ No ☐Unknown<br>Age(s) at Diagnosis:	☐ Yes ☐ No<br>☐ Unknown<br>Type:<br>Age at Diagnosis:	☐ Never<br>☐ Occasional<br>☐ Regular<br>☐ Unknown<br># Years Smoked:<br># Cigarettes/Day:	☐Yes ☐ No<br>Age At Death:<br>Cause of Death:
		**Pancreas Cancer:** ☐ Yes ☐ No ☐Unknown<br>**Diabetes:** ☐ Yes ☐ No ☐Unknown<br>**Pancreatitis**: ☐ Yes ☐ No ☐Unknown<br>Age(s) at Diagnosis:	☐ Yes ☐ No<br>☐ Unknown<br>Type:<br>Age at Diagnosis:	☐ Never<br>☐ Occasional<br>☐ Regular<br>☐ Unknown<br># Years Smoked:<br># Cigarettes/Day:	☐Yes ☐ No<br>Age At Death:<br>Cause of Death:

THANK YOU VERY MUCH!

Please do not hesitate to call us on 02 83822061 if you have any questions.
