# Supplementary material for: Five-year psychological impact and surveillance compliance in the Australian Pancreatic Cancer Screening Program
Source: Fam Cancer. 2026 Apr 28;25(2):46. doi: 10.1007/s10689-026-00557-0 (PMC13124842; doi:10.1007/s10689-026-00557-0)
Supplement: Supplementary file 2 — Supplementary Material 2. 1-month Questionnaire [file 10689_2026_557_MOESM2_ESM.pdf]

FOLLOW UP QUESTIONNAIRE (1 month)

Name: ____________________________ **Date: _______________**

A. Impact of events scale

Below is a list of comments made by people about being at risk for pancreatic cancer. Please tick a box to indicate how frequently these comments were true for you during *the last seven days.*

		Not at all	Rarely	Sometimes	Often
1.	I thought about it when I didn't mean to	❑	❑	❑	❑
2.	I avoided letting myself get upset when I thought about or was reminded of it	❑	❑	❑	❑
3.	I tried to remove it from my memory	❑	❑	❑	❑
4.	I had trouble falling asleep or staying asleep because of pictures or thoughts about it that came into my mind	❑	❑	❑	❑
5.	I had waves of strong feelings about it	❑	❑	❑	❑
6.	I had dreams about it	❑	❑	❑	❑
7.	I stayed away from reminders of it	❑	❑	❑	❑
8.	I felt as if it wasn't real	❑	❑	❑	❑
9.	I tried not to talk about it	❑	❑	❑	❑
10.	Pictures popped up into my mind	❑	❑	❑	❑
11.	Other things kept making me think about it	❑	❑	❑	❑
12.	I was aware that I still had a lot of feelings about it, but I didn't deal with them	❑	❑	❑	❑
13.	I tried not to think about it	❑	❑	❑	❑
14.	Any reminder brought back feelings about it	❑	❑	❑	❑
15.	My feelings were sort of numb	❑	❑	❑	❑

Office use only:
Subtotal I _____
Subtotal A _____
Total Score _____

B. Personal Consequences Questionnaire

We would like to know your experiences of the screening procedure and your thoughts and feelings about pancreatic cancer.

1. Over the ***last week*** have you experienced the following things because of thoughts and feelings about *pancreatic cancer*:

	Not at all	Rarely	Some of the time	Quite a lot of the time
a. Had trouble sleeping	0	1	2	3
b. Experienced a change in appetite	0	1	2	3
c. Been unhappy or depressed	0	1	2	3
d. Been scared and panicky	0	1	2	3
e. Felt nervous or strung up	0	1	2	3
f. Felt under strain	0	1	2	3
g. Found you have been keeping things from those who are close to you	0	1	2	3
h. Found yourself taking things out on other people	0	1	2	3
i. Found yourself noticeable withdrawing from those who are close to you	0	1	2	3
j. Had difficulty doing things around the house which you normally do	0	1	2	3
k. Had difficulty meeting work or other commitments	0	1	2	3
l. Feeling worried about your future	0	1	2	3

2. **All things considered, would you say your experiences at the screening procedure have caused any of the following**:

	Not at all	Rarely	Some of the time	Quite a lot of the time
a. A sense of reassurance that you do not have pancreatic cancer	0	1	2	3
b. Feeling more relaxed	0	1	2	3
c. Improved relationship with friends or relations	0	1	2	3
d. Feeling more able to do things which you normally do	0	1	2	3
e. Feeling more able to meet your home and/or work responsibilities	0	1	2	3
f. Feeling more hopeful about the future	0	1	2	3
g. Feeling less anxious about pancreatic cancer	0	1	2	3
h. Getting on better with those around you	0	1	2	3
i. Been sleeping better	0	1	2	3
j. A greater sense of well being	0	1	2	3

C. We would like to know your experience with the genetic counselling for pancreas cancer risk.

These questions refer to your consult at ______________________________

	Strongly Agree	*Partially Agree*	*Neutral*	*Partially Disagree*	*Strongly Disagree*
1. I knew beforehand that I would receive genetic counselling as part of my research visit.	1	2	3	4	5
2. Before genetic counselling, I had already read or heard a fair amount about hereditary pancreas cancer.	1	2	3	4	5
3. The genetic counselling session was helpful to me	1	2	3	4	5
4. I would have preferred to have only the endoscopy procedure and not genetic counselling	1	2	3	4	5
5. I would have preferred more information about hereditary pancreas cancer.	1	2	3	4	5
6. Scientists do not currently know enough about hereditary pancreas cancer to help me.	1	2	3	4	5

	Strongly Agree	*Partially Agree*	*Neutral*	*Partially Disagree*	*Strongly Disagree*
7. I would recommend genetic counselling for pancreas cancer to a friend or relative with a family history of pancreas cancer.	1	2	3	4	5
8. The genetic information was too complex	1	2	3	4	5
9. The written summary of the visit was useful	1	2	3	4	5
10. I will share the written summary with my family members	1	2	3	4	5
11. I think genetic counselling for pancreas cancer is helpful, even if the "pancreas cancer gene" has not been found	1	2	3	4	5
12. If the "pancreas cancer gene" were found, I would want to be tested for it	1	2	3	4	5
13. I do not think genetic counselling should be offered for pancreas cancer until the "pancreas cancer gene" has been found.	1	2	3	4	5
14. I would be interested in another genetic counselling session when more information is learned about pancreas cancer	1	2	3	4	5
15. I think that the pancreas cancer in my family is caused by a gene mutation.	1	2	3	4	5
16. I think I inherited a gene mutation that causes pancreas cancer.	1	2	3	4	5
(for participants who have NOT previously had genetic testing related to pancreas cancer): 17. Even though the "pancreas cancer gene" has not been found, I still want to get genetic testing for one or more of the syndromes discussed during genetic counselling.	1	2	3	4	5
(for participants who HAVE previously had genetic testing related to pancreas cancer): 18. I am glad that I had genetic testing for cancer risk.	1	2	3	4	5
19. I do not feel that current genetic testing is likely to help me	1	2	3	4	5

20. The most useful part, if any, of genetic counselling was:

___

___

___

21. The least useful part, if any, of genetic counselling was:

___

___

___

Thank you for your time. You have finished this questionnaire!

Please return the questionnaire in the enclosed reply paid envelope, and post it within the next seven days, if possible
