# Supplementary material for: Five-year psychological impact and surveillance compliance in the Australian Pancreatic Cancer Screening Program
Source: Fam Cancer. 2026 Apr 28;25(2):46. doi: 10.1007/s10689-026-00557-0 (PMC13124842; doi:10.1007/s10689-026-00557-0)
Supplement: Supplementary file 3 — Supplementary Material 3. 1-year and 5-year Questionnaire [file 10689_2026_557_MOESM3_ESM.pdf]

FOLLOW UP QUESTIONNAIRE (1 - 5 years)

Name: ______________________________ Date: ______________

A. Impact of events scale

Below is a list of comments made by people about being at risk for pancreatic cancer. Please tick a box to indicate how frequently these comments were true for you during *the last seven days.*

		Not at all	Rarely	Sometimes	Often
1.	I thought about it when I didn't mean to	❑	❑	❑	❑
2.	I avoided letting myself get upset when I thought about or was reminded of it	❑	❑	❑	❑
3.	I tried to remove it from my memory	❑	❑	❑	❑
4.	I had trouble falling asleep or staying asleep because of pictures or thoughts about it that came into my mind	❑	❑	❑	❑
5.	I had waves of strong feelings about it	❑	❑	❑	❑
6.	I had dreams about it	❑	❑	❑	❑
7.	I stayed away from reminders of it	❑	❑	❑	❑
8.	I felt as if it wasn't real	❑	❑	❑	❑
9.	I tried not to talk about it	❑	❑	❑	❑
10.	Pictures popped up into my mind	❑	❑	❑	❑
11.	Other things kept making me think about it	❑	❑	❑	❑
12.	I was aware that I still had a lot of feelings about it, but I didn't deal with them	❑	❑	❑	❑
13.	I tried not to think about it	❑	❑	❑	❑
14.	Any reminder brought back feelings about it	❑	❑	❑	❑
15.	My feelings were sort of numb	❑	❑	❑	❑

Office use only:
Subtotal I _____
Subtotal A __
Total Score _____

B. Personal Consequences Questionnaire

We would like to know your experiences of the screening procedure and your thoughts and feelings about pancreatic cancer.

1. Over the ***last week*** have you experienced the following things because of thoughts and feelings about *pancreatic cancer*:

	Not at all	Rarely	Some of the time	Quite a lot of the time
a. Had trouble sleeping	0	1	2	3
b. Experienced a change in appetite	0	1	2	3
c. Been unhappy or depressed	0	1	2	3
d. Been scared and panicky	0	1	2	3
e. Felt nervous or strung up	0	1	2	3
f. Felt under strain	0	1	2	3
g. Found you have been keeping things from those who are close to you	0	1	2	3
h. Found yourself taking things out on other people	0	1	2	3
i. Found yourself noticeable withdrawing from those who are close to you	0	1	2	3
j. Had difficulty doing things around the house which you normally do	0	1	2	3
k. Had difficulty meeting work or other commitments	0	1	2	3
l. Feeling worried about your future	0	1	2	3

2. **All things considered, would you say your experiences at the screening procedure have caused any of the following**:

	Not at all	Rarely	Some of the time	Quite a lot of the time
a. A sense of reassurance that you do not have pancreatic cancer	0	1	2	3
b. Feeling more relaxed	0	1	2	3
c. Improved relationship with friends or relations	0	1	2	3
d. Feeling more able to do things which you normally do	0	1	2	3
e. Feeling more able to meet your home and/or work responsibilities	0	1	2	3
f. Feeling more hopeful about the future	0	1	2	3
g. Feeling less anxious about pancreatic cancer	0	1	2	3
h. Getting on better with those around you	0	1	2	3
i. Been sleeping better	0	1	2	3
j. A greater sense of well being	0	1	2	3

Thank you for your time. You have finished this questionnaire!

Please return the questionnaire in the enclosed reply paid envelope, and post it within the next seven days, if possible.
