# Supplementary material for: Five-year psychological impact and surveillance compliance in the Australian Pancreatic Cancer Screening Program
Source: Fam Cancer. 2026 Apr 28;25(2):46. doi: 10.1007/s10689-026-00557-0 (PMC13124842; doi:10.1007/s10689-026-00557-0)
Supplement: Supplementary file 4 — Supplementary Material 4. Table S1 and Table S2 Estimated Marginal Means on the latent scale, using generalized linear mixed model analyses [file 10689_2026_557_MOESM4_ESM.pdf]

Five-year psychological impact and surveillance compliance in the Australian Pancreatic Cancer Screening Program, Familial Cancer, Tanya M Dwarte, David K E Chan, Nick Olsen, David B Williams, Anthony J Gill; Alina Stoita

Corresponding Author: Dr Alina Stoita, Department of Gastroenterology, St Vincent's Hospital, Darlinghurst, NSW Australia 2010. Email: alina.stoita@svha.org.au

Table S1 Estimated Marginal Means for Impact of Event Scale scores, on the latent scale, using generalized linear mixed model analyses

Impact of Event Scale	Estimated Marginal Mean $\hat{\mu}$ (95% HPD CI)	
**Intrusion**	**Available case analysis**	**Multiple imputation**
Baseline	8.19 (4.59 to 11.83)	7.69 (4.20 to 11.17)
1-Month	5.02 (0.61 to 8.25)	4.07 (0.53 to 7.62)
1-Year	5.46 (1.29 to 8.80)	3.86 (0.32 to 7.39)
5-Years	6.32 (2.13 to 9.87)	4.23 (0.67 to 7.80)
**Avoidance**		
Baseline	9.22 (3.68 to 14.11)	8.75 (3.87 to 13.63)
1-Month	6.19 (0.73 to 11.20)	5.29 (0.37 to 10.21)
1-Year	6.58 (1.12 to 11.93)	4.65 (-0.26 to 9.56)[a]
5-Years	8.58 (2.95 to 13.70)	5.59 (0.62 to 10.56)
**Total**		
Baseline	18.65 (9.68 to 25.60)	17.72 (10.48 to 24.96)
1-Month	12.56 (4.32 to 20.16)	10.96 (3.66 to 18.27)
1-Year	13.58 (5.29 to 20.90)	10.35 (3.07 to 17.63)
5-Years	16.17 (7.73 to 23.64)	11.57 (4.22 to 18.92)

[a] 95% HDP CI exceeded the lower limit

Abbreviations: CI, confidence interval; HPD, highest posterior density

Table S2 Estimated Marginal Means for Negative and Positive Psychological Consequences Questionnaire scores, on the latent scale, using generalized linear mixed model analyses

Psychological Consequences Questionnaire	Estimated Marginal Mean $\hat{\mu}$ (95% HPD CI)			
	Negative PCQ		Positive PCQ	
**Emotional**	**Available case analysis**	**Multiple imputation**	**Available case analysis**	**Multiple imputation**
Baseline	1.36 (-0.88 to 3.61)[a]	1.48 (-0.52 to 3.47)[a]	-	-
1-Month	0.60 (-2.13 to 2.41)[a]	0.40 (-1.62 to 2.43)[a]	10.97 (8.28 to 13.99)	10.82 (8.67 to 12.97)
1-Year	0.88 (-1.50 to 3.01)[a]	0.26 (-1.77 to 2.29)[a]	12.11 (9.40 to 15.17)[c]	12.08 (9.93 to 14.23)
5-Years	1.25 (-1.17 to 3.40)[a]	0.07 (-1.98 to 2.11)[a]	13.84 (11.06 to 17.01)[c]	13.35 (11.12 to 15.59)[c]
**Physical**				
Baseline	-1.66 (-4.66 to 1.09)[b]	-1.59 (-3.81 to 0.63)[b]	-	-
1-Month	-2.30 (-5.59 to 0.48)[b]	-2.65 (-4.92 to -0.39)[b]	3.38 (-0.17 to 7.14)[a]	3.23 (0.32 to 6.15)
1-Year	-2.05 (-5.61 to 0.53)[b]	-2.60 (-4.85 to -0.34)[b]	3.33 (-0.12 to 7.18)[a]	3.37 (0.46 to 6.28)
5-Years	-1.43 (-4.87 to 1.25)[b]	-2.82 (-5.12 to -0.52)[b]	5.81 (1.89 to 9.50)[c]	5.89 (2.92 to 8.87)
**Social**				
Baseline	-1.95 (-9.55 to 0.24)[b]	-1.92 (-3.99 to 0.16)[b]	-	-
1-Month	-2.27 (-9.65 to 0.13)[b]	-2.55 (-4.64 to -0.47)[b]	1.69 (-0.72 to 4.10)[a]	1.72 (-0.34 to 3.77)[a]
1-Year	-1.82 (-9.27 to 0.59)[b]	-2.26 (-4.38 to -0.15)[b]	1.72 (-0.58 to 4.35)[a]	1.95 (-0.11 to 4.01)[a]
5-Years	-1.37 (-8.87 to 0.98)[b]	-2.83 (-5.04 to -0.62)[b]	3.35 (1.03 to 5.98)	3.60 (1.54 to 5.66)
**Total**				
Baseline	2.85 (-1.76 to 7.00)[a]	3.06 (-0.69 to 6.81)[a]	-	-
1-Month	1.44 (-3.34 to 5.62)[a]	1.04 (-2.74 to 4.81)[a]	18.28 (14.03 to 23.14)	17.57 (13.73 to 21.41)
1-Year	2.04 (-2.10 to 7.08)[a]	1.06 (-2.73 to 4.85)[a]	19.27 (14.89 to 23.78)	18.82 (14.95 to 22.68)
5-Years	3.08 (-1.93 to 6.94)[a]	0.52 (-3.34 to 4.38)[a]	23.27 (18.62 to 28.00)	22.27 (18.35 to 26.18)

[a] 95% HDP CI exceeded the lower limit
[b] Estimated marginal mean and 95% HPD CI exceeded the lower limit
[c] 95% HPD CI exceeded the upper limit

Abbreviations: CI, confidence interval; EUS, endoscopic ultrasound; FPC, Familial Pancreatic Cancer; HPD, highest posterior density; MRI, Magnetic resonance imaging; PDAC, pancreatic ductal adenocarcinoma; PHx, personal history
